# Mesenchymal stem cell therapies for ARDS: translational promise and challenges

**DOI:** 10.1186/s13287-025-04614-w

**Published:** 2025-09-26

**Authors:** Fengyun Wang, Chengzhi Xie, Xiaozhi Wang

**Affiliations:** 1 Department of Critical Care Medicine, Second Affiliated Hospital of Hainan Medical University, Haikou, China; 2 NHC Key Laboratory of Tropical Disease Control, Hainan Medical University, Haikou, China

**Keywords:** Mesenchymal stem cells, Acute respiratory distress syndrome, Cell-free therapies, Translational research, Immunomodulation

## Abstract

Over the past decade, global investigations have rigorously assessed the safety and therapeutic potential of mesenchymal stem cells (MSCs) in managing acute respiratory distress syndrome (ARDS). MSCs, obtained from sources like bone marrow, adipose tissue, and umbilical cord, exert therapeutic effects in ARDS primarily through complex paracrine mechanisms, including anti-inflammatory, immunoregulatory, pro-reparative, antioxidant, antimicrobial, and mitochondrial transfer functions. Preclinical studies have consistently demonstrated significant therapeutic benefits. Clinical trials have further confirmed a favorable safety profile, with no significant infusion-related toxicity or serious adverse events observed even at higher doses (up to 10 × 10⁶ cells/kg) or following multiple administrations. Yet, while some early-phase clinical trials have not conclusively demonstrated a significant reduction in mortality among ARDS patients, multiple studies note diminished inflammatory biomarkers, enhanced markers of endothelial and epithelial repair (e.g., angiopoietin-2), and suggestive benefits in subgroups like younger patients or those receiving higher doses of viable cells. MSC-derived therapies, particularly extracellular vesicles and conditioned medium, represent promising “cell-free” strategies that may overcome limitations associated with live-cell therapy. Despite encouraging progress, clinical translation faces challenges, including optimizing cell sources, preparation, dosing, delivery, and developing robust potency assays. Future research should prioritize large, high-quality randomized trials to confirm efficacy across various ARDS etiologies and clinical phenotypes, evaluate repeat dosing, and explore innovative strategies such as gene modification, cellular preconditioning, and combination therapies. Collectively, MSCs and their derivatives hold substantial potential for ARDS treatment, though their widespread application requires further validation and a deeper understanding of their interactions with the complex ARDS microenvironment.

**Supplementary Information:**

The online version contains supplementary material available at 10.1186/s13287-025-04614-w.

## Introduction

Acute respiratory distress syndrome (ARDS) is a life-threatening condition characterized by disruption of the alveolar-capillary barrier, pulmonary edema, severe hypoxemia, and reduced lung compliance, precipitated by diverse direct or indirect insults such as pneumonia, sepsis, trauma, or viral infections [1]. Despite improvements in lung-protective ventilation strategies (e.g., low tidal volume ventilation) and supportive therapies (e.g., extracorporeal membrane oxygenation [ECMO] and prone positioning), the mortality rate of ARDS remains high (30% – 40%), and no specific pharmacological treatment has been demonstrated to be effective [2]. Consequently, developing novel therapeutic approaches to improve ARDS outcomes is of critical clinical importance.

Mesenchymal stem cells (MSCs), also referred to as mesenchymal stromal cells, are adult stem cells characterized by their multilineage differentiation potential, self-renewal capability, and potent immunomodulatory functions [3]. They can be isolated from multiple tissues, including bone marrow (BM), adipose tissue (AT), umbilical cord (UC), and placenta (PL) [4]. Over the past decade, researchers worldwide have conducted extensive preclinical and clinical studies to evaluate the safety and efficacy of MSC-based therapies for the treatment of ARDS [5, 6]. Extensive preclinical evidence has demonstrated that MSCs significantly reduce lung injury and improve survival in various ARDS animal models via multiple mechanisms, including anti-inflammatory, anti-apoptotic, epithelial, and endothelial repair, enhanced bacterial clearance, and antioxidant activity [7–11]. These encouraging preclinical findings have propelled clinical translation; early-phase trials suggest MSC administration is safe and potentially improves outcomes in moderate-to-severe ARDS patients [12].

MSC therapy is primarily indicated for moderate-to-severe ARDS caused by diverse etiologies, particularly in critically ill patients with multi-organ failure, with inclusion criteria often based on partial pressure of oxygen to fraction of inspired oxygen (PaO_2_/FiO_2_) ratios (e.g., < 200 mmHg) and inflammatory biomarker levels [5, 10, 12, 13]. Clinical trials typically target the acute phase of ARDS (within 96 h of diagnosis) to maximize the benefit of early intervention in controlling inflammation and lung damage [14]. However, MSC therapy remains investigational, requiring further evidence to confirm its efficacy and safety. Here, we summarize the mechanisms of MSC, their sources and delivery strategies, clinical outcomes, cell-free derivatives, efficacy across ARDS subtypes, and key challenges for future translation.

### Therapeutic mechanisms of MSCs in ARDS

**Fig. 1:**
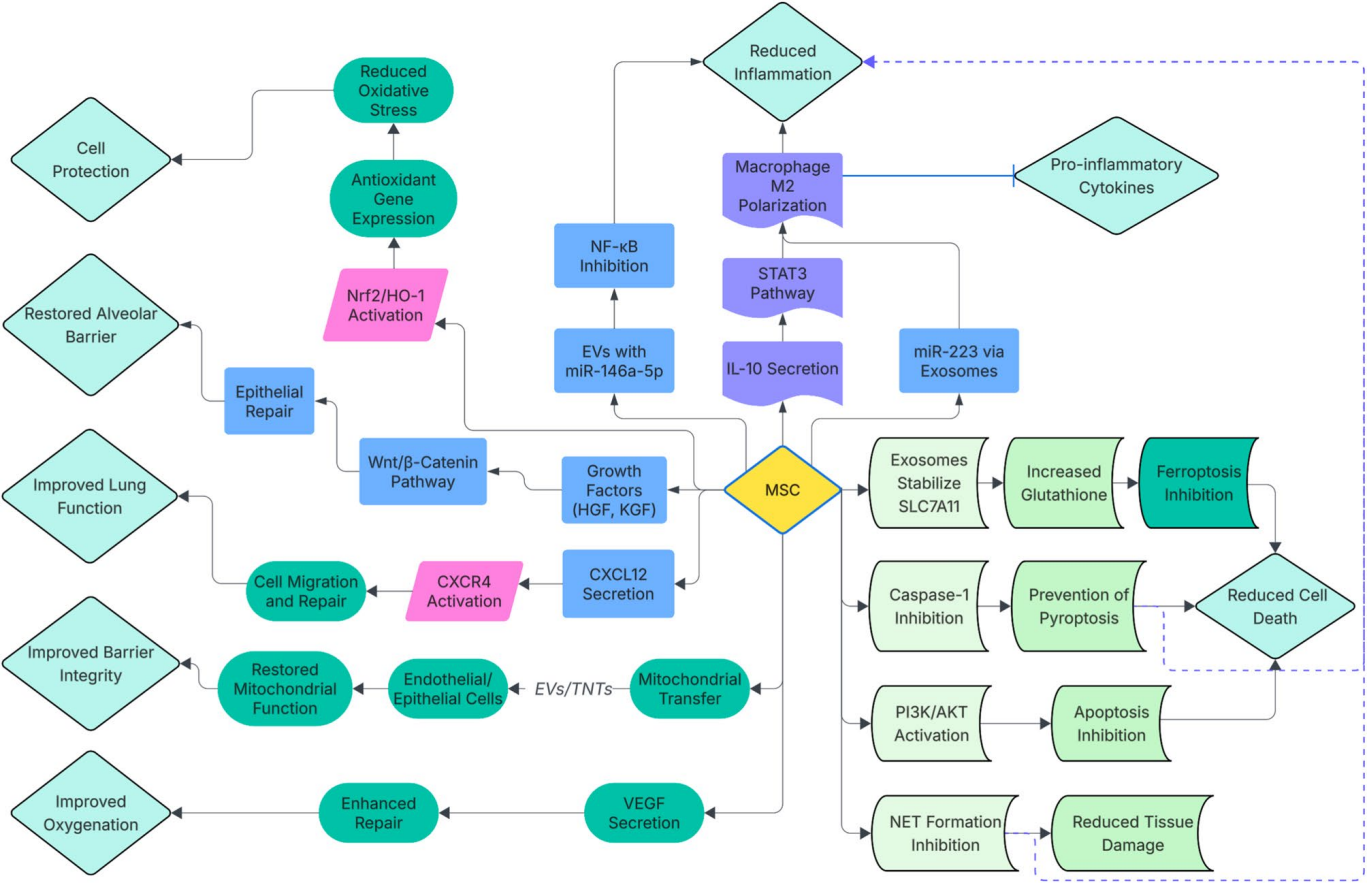
Illustration of the multifaceted mechanisms by which MSCs exert therapeutic effects in ARDS and ALI. MSCs mediate immunomodulation by interacting with immune cells to attenuate inflammation and promote tissue repair through modulation of epithelial and endothelial cells. They enhance alveolar barrier integrity and cellular function via antioxidant activity, inhibition of ferroptosis, and mitochondrial transfer (via EVs or TNTs). These actions collectively reduce cell death and improve oxygenation in ARDS/ALI. Key molecular mediators include cytokines (e.g., IL-10), chemokines (e.g., CXCL12), microRNAs (e.g., miR-146a-5p), growth factors (e.g., HGF), transcription factors (e.g., STAT3), cell death regulators (e.g., caspase-1), and receptors (e.g., CXCR4) [32, 46, 48, 52, 82, 108, etc]. ARDS: Acute respiratory distress syndrome; ALI: Acute Lung Injury༛MSC: Mesenchymal Stem Cell༛IL-10:Interleukin-10༛CXCL12:C-X-C Motif Chemokine Ligand 12༛miR-146a-5p: microRNA-146a-5p༛HGF: Hepatocyte Growth Factor༛STAT3:Signal Transducer and Activator of Transcription 3༛Caspase-1:Cysteine-Aspartate Protease 1༛CXCR4:C-X-C Motif Chemokine Receptor 4

The therapeutic mechanisms of MSCs in ARDS are multifaceted and predominantly mediated through paracrine effects rather than long-term engraftment and differentiation at injury sites [15]. The evidence-based mechanistic network centered on MSCs and their therapeutic role in ARDS is depicted in Fig. 1.

#### Immunomodulation

MSCs respond to inflammatory cues within the damaged microenvironment by releasing a diverse repertoire of soluble mediators and extracellular vesicles (EVs) that modulate both innate and adaptive immune responses. They suppress the expression of pro-inflammatory cytokines such as TNF-α, IL-1β, IL-6, and MIP-2 [16–18], while promoting anti-inflammatory mediators, notably IL-10 [19, 20]. MSCs also shift macrophages from M1 to M2 phenotypes [13, 21, 22], and balance Tregs against Th17 cells to mitigate inflammation [23].

At the signaling level, MSCs exert critical immunomodulatory effects in ARDS by targeting multiple pathways. They significantly attenuate excessive inflammation by inhibiting pro-inflammatory cascades, including the nuclear factor κB (NF-κB) pathway, thereby reducing the production of pro-inflammatory cytokines [24, 25]. MSCs also modulate the receptor for advanced glycation end-products (RAGE) [24] and Toll-like receptor 4 (TLR4) [17, 26], both of which play pivotal roles in amplifying inflammatory responses to tissue damage and pathogens, thus limiting further lung injury. Concurrently, MSCs bolster cellular defense and repair mechanisms. For instance, engagement of the nuclear factor erythroid 2-related factor 2 (Nrf2) pathway underpins broad cytoprotective effects through its potent anti-inflammatory and antioxidant actions [17, 25, 27]. These multifaceted actions are complemented by the engagement with the cholinergic anti-inflammatory pathway [28], the secretion of anti-inflammatory mediators like tumor necrosis factor-stimulated gene-6 (TSG-6) [29], and the regulation of soluble TNF receptor 2 (sTNFR2) [30], all contributing to a more balanced immune response and promoting tissue protection in the ARDS lung.

#### Enhanced alveolar-capillary barrier repair

Beyond immunomodulation, MSCs contribute to alveolar-capillary barrier repair. Studies have shown that MSCs can reduce pulmonary vascular permeability and alleviate pulmonary edema [13, 31], partly through the secretion of growth factors such as angiopoietin-1 (Ang-1) [32], keratinocyte growth factor (KGF) [33], hepatocyte growth factor (HGF) [34], and vascular endothelial growth factor (VEGF) [35]. These factors support the regeneration of alveolar epithelial and endothelial cells, restore barrier integrity, and stabilize the vascular structure. MSCs also possess anti-fibrotic properties, as demonstrated by their ability to reduce collagen deposition and attenuate lung structural abnormalities in bleomycin- and paraquat-induced pulmonary fibrosis models [36–38]. Moreover, MSCs safeguard pulmonary cells by inhibiting apoptotic signaling, particularly by regulating the Bax/Bcl-2 balance [39, 40].

#### Reduced oxidative stress

MSCs alleviate oxidative stress by decreasing the levels of malondialdehyde (MDA) and reactive oxygen species (ROS), and by enhancing the activities of superoxide dismutase (SOD) and glutathione (GSH) [25, 41, 42]. In infectious ARDS, MSCs also promote bacterial clearance, which contributes to reduced pulmonary bacterial burden and improved host defense [43, 44]. Moreover, the Hippo pathway contributes to these antioxidant effects by augmenting MSC antioxidant capabilities [45], further protecting lung tissue.

#### Mitochondrial transfer

MSCs are capable of transferring functional mitochondria to various injured pulmonary cell types, including alveolar epithelial cells, endothelial cells, and macrophages [46]; this process directly impacts ARDS outcomes through multiple mechanisms [33, 47]. A primary effect of this transfer is the restoration of cellular bioenergetic function. By delivering healthy mitochondria, typically via extracellular vesicles (EVs) [46] or tunneling nanotubes (TNTs) [48], MSCs replenish ATP supplies, enhance oxidative phosphorylation, and improve oxygen consumption rates in recipient cells, all of which are critical for cellular repair and survival in the face of ARDS-induced metabolic stress [49–51]. This bioenergetic rescue is crucial for maintaining alveolar-capillary barrier integrity, a key aspect compromised in ARDS [50, 52].

Beyond direct bioenergetic recovery, mitochondrial transfer also exerts significant anti-apoptotic effects. The provision of functional mitochondria modulates apoptosis-related pathways, such as by increasing anti-apoptotic Bcl-2 levels and preserving mitochondrial membrane potential, thereby protecting lung cells from programmed cell death [53–55]. For instance, MSC-derived exosomes facilitate mitochondrial transfer to alveolar macrophages, enhancing not only their bioenergetics but also their homeostasis, and phagocytic capacity, while shifting them towards an anti-inflammatory phenotype, all contributing to the resolution of inflammation and tissue repair [46, 48, 53]. Furthermore, mitochondrial transfer to pulmonary endothelial cells can activate metabolic pathways like the TCA cycle, promoting endothelial proliferation, the release of pro-angiogenic factors, and ultimately enhancing vascular regeneration, which is vital for repairing the damaged lung vasculature in ARDS [51, 52].

#### Other mechanisms

Emerging evidence indicates MSCs may also mitigate lung inflammation and injury by modulating gut microbiota, suggesting a regulatory role via the lung-gut axis [26, 56, 57]. Substantial evidence indicates that MSC engraftment in lung tissue is generally low and short-lived following transplantation [8]. Thus, their therapeutic impact primarily stems from paracrine signaling, involving the secretion of soluble factors like IL-10 and transforming growth factor-beta (TGF-β), alongside EVs, which deliver anti-inflammatory signals, boost macrophage phagocytosis, and promote tissue regeneration [15–17, 58].

#### Donor age and host microenvironment

The therapeutic effects of MSCs are not only determined by their inherent properties but also critically influenced by donor age and the recipient’s inflammatory microenvironment. MSCs derived from younger donors typically demonstrate superior regenerative potential compared to those from aged sources. MSCs from older individuals often exhibit signs of cellular senescence, including shortened telomeres, reduced proliferative capacity, and diminished differentiation potential [59–61]. This age-related decline in MSC “fitness” is also associated with increased oxidative damage, higher levels of reactive oxygen species (ROS), and upregulation of senescence-related genes like p21 and p53 [62]. In addition, senescent MSCs frequently shift toward a pro-inflammatory senescence-associated secretory phenotype (SASP), characterized by reduced secretion of reparative paracrine factors such as HGF and IL-10, and increased release of inflammatory mediators, thereby impairing their immunomodulatory and reparative capacities [63–65].

The host microenvironment also plays a crucial role in regulating MSC function. A highly inflammatory milieu—particularly one with elevated IL-6—elevated IL-6, can compromise MSC immunoregulatory activity by disrupting key intracellular signaling pathways such as STAT3 or NF-κB [64, 66]. Conversely, a less hostile and more balanced immune environment, often observed in younger patients, may facilitate MSC survival, retention, and therapeutic efficacy. This dynamic interplay between donor cell characteristics and recipient immunologic status highlights the importance of considering both intrinsic and extrinsic factors when optimizing MSC-based therapies for ARDS.

### MSC sources and their characteristics

MSCs can be sourced from various tissues, with BM-MSCs, AT-MSCs, UC-MSCs, PL-MSCs, and menstrual blood (Menstrual blood-MSC) being commonly studied in preclinical and clinical settings [4]. Each source presents unique advantages and limitations regarding availability, proliferative capacity, immunomodulatory potential, and ethical considerations. A comparative overview of the characteristics and therapeutic features of MSCs from different sources in ARDS is presented in Table 1.

**Table 1:** Comparison of MSC sources for ARDS treatment

MSC Source	Yield	Immunogenicity	Clinical Efficacy	Scalability	Reference
BM-MSCs	Moderate; limited by invasive bone marrow aspiration, requires in vitro expansion	Moderate; requires HLA matching to reduce rejection risk	Improves oxygenation and reduces inflammation in ARDS; mixed results in clinical trials (e.g., 28-day mortality 30% vs. 15%, *P* = 0.58)	Low; invasive collection and complex expansion limit large-scale production	[13, 16, 79]
AT-MSCs	High; abundant adipose tissue via liposuction, high cell yield per procedure	Low; minimal rejection risk in allogeneic use	Reduces inflammation and improves lung function in preclinical and early clinical studies	High; minimally invasive, standardized liposuction enables large-scale production	[68, 69]
UC-MSCs	High; umbilical cord tissue readily available, efficient in vitro expansion	Low; suitable for allogeneic therapy due to immune-privileged status	Significantly improves survival in COVID-19 ARDS (91% vs. 42%, *P* = 0.015) and reduces inflammatory cytokines	High; non-invasive, abundant source, supports standardized production	[10, 33, 70]
PL-MSCs	High; plentiful placental tissue, high cell yield per extraction	Low; immune-privileged, low rejection risk	Suppresses inflammation and promotes lung repair in early clinical studies	High; abundant tissue source, feasible for large-scale production	[73–75]

Abbreviations: MSC Mesenchymal Stromal Cells; BM-MSC Bone Marrow-Derived MSCs; AT-MSC Adipose Tissue-Derived MSCs; UC-MSCs Umbilical Cord-Derived MSCs; PL-MSCs Placenta-Derived MSCs; ARDS Acute Respiratory Distress Syndrome; HLA Human Leukocyte Antigen; IV Intravenous; OR Odds Ratio

#### BM-MSCs

BM-MSCs are among the most extensively studied MSC types and are known for their potent immunomodulatory properties [16]. Hao et al. reported that BM-MSCs significantly attenuated LPS-induced pulmonary edema and inflammation, although their effects on reducing alveolar protein leakage were limited [13]. The START clinical trials confirmed that a single intravenous infusion of BM-MSCs at doses up to 10 × 10⁶ cells/kg was well tolerated [67]. Nevertheless, BM-MSCs face challenges, including an invasive harvesting procedure and diminished yield and proliferative capacity with advancing donor age.

#### AT-MSCs

AT-MSCs can be harvested in large quantities through liposuction; however, they exhibit greater heterogeneity and tend to have shorter pulmonary retention times in ARDS models [68]. Zheng et al. reported that treatment with AD-MSCs resulted in decreased levels of surfactant protein-D (SP-D), yet primary clinical outcomes showed no significant improvement [69].

#### UC-MSCs

UC-MSCs, sourced from abundant, ethically uncontentious materials (often considered medical waste), exhibit youthful cellular vigor, robust proliferation, and low immunogenicity, rendering them highly appealing for clinical studies [31, 70–72]. Lanzoni et al. reported that treatment with UC-MSCs in patients with COVID-19-associated ARDS led to improved survival and decreased levels of inflammatory cytokines [10]. Both in vitro and animal studies have indicated that UC-MSCs exhibit therapeutic efficacy comparable to BM-MSCs [43].

#### PL-MSCs

PL-MSCs, which are similarly abundant, highly proliferative, and exhibit low immunogenicity, demonstrate potent anti-inflammatory properties via IL-10/STAT3/NLRP3 axis [73] and are considered suitable for standardized production. Xu et al. demonstrated that PL-MSCs attenuate LPS-induced increases in endothelial permeability and reduce pulmonary injury [74]. A phase I clinical trial in patients with COVID-19-associated ARDS confirmed the safety of intravenous infusion of PL-MSCs; however, no significant differences in therapeutic efficacy were observed compared to the control group [75].

### Administration and clinical outcomes

Intravenous (IV) infusion remains the predominant clinical delivery method due to its practicality, yet it encounters a “first-pass effect,” where many cells are temporarily sequestered in the pulmonary vasculature before redistribution or clearance [68]. Intratracheal (IT) administration and nebulization allow direct delivery to the lungs, potentially enhancing local therapeutic effects. Numerous preclinical studies affirm their feasibility and efficacy [16, 17, 76, 77], though their clinical safety and effectiveness await further validation. Intrapleural administration has also been investigated and is believed to exert therapeutic effects primarily through paracrine mechanisms [15].

Preclinical studies have explored diverse dosing regimens, whereas clinical trials typically administer doses between 1 × 10⁶ and 10 × 10⁶ cells per kilogram [6, 78], with the START trial confirming IV tolerability up to 10 × 10⁶ cells/kg [79]. However, higher doses may increase the risk of adverse events such as fever and coagulopathy [80, 81]. Most studies favor single doses [69, 79], but some trials have evaluated multiple dosing regimens (e.g., on days 0 and 3, or two to four infusions), demonstrating safety and suggesting possible benefits in survival and pulmonary function [82–86].

The dose-response relationship in MSC therapy for ARDS reveals conflicting evidence. While the START 2a trial (10 × 10⁶ cells/kg) found no mortality reduction (30% vs. 15%, *P* = 0.58), it noted improved oxygenation and reduced endothelial injury with higher viable cell counts [79]. In contrast, Lanzoni et al. (100 × 10⁶ cells, two doses) observed significant survival benefits (91% vs. 42%, *P* = 0.015) [10]. Preclinical data suggest higher doses enhance efficacy [87, 88]. Variability may stem from cell viability [12], host microenvironment (e.g., inflammation, comorbidities) [66], and ARDS heterogeneity. Optimizing cell viability and tailoring dosing strategies to individual patient contexts may improve therapeutic outcomes. Patient-stratified trials are warranted to clarify dosing strategies.

Preclinical evidence suggests that early administration of MSCs—within 24 h after injury—yields superior outcomes compared to delayed administration (e.g., after 48 h), including enhanced anti-inflammatory effects and improved pulmonary retention [68, 80, 88]. In clinical trials, MSC therapy is typically initiated within 48 to 96 h following ARDS diagnosis [79]. Sánchez-Guijo et al. observed a higher rate of extubation in patients who received treatment within 48 h [83]. The optimal timing for MSC intervention—whether during the early inflammatory phase or the later reparative phase—remains unclear and warrants further investigation [89].

Following IV infusion, MSCs primarily localize to the lungs, where they exhibit transient retention [8]. Lung injury has been shown to enhance the homing capacity of MSCs through chemokine-mediated mechanisms [90, 91], though their limited post-transplantation survival markedly constrains their long-term therapeutic efficacy [92].

Preclinical studies consistently show that MSC therapy lowers mortality in ARDS animal models [11]. Clinical trial outcomes have been heterogeneous. Open-label studies report promising results [82, 85, 93], but rigorous RCTs like START 2a have not confirmed significant mortality reductions [79]. Meta-analyses have suggested that MSC therapy may reduce overall mortality in patients with ARDS, including those with COVID-19-associated ARDS. However, substantial heterogeneity among studies necessitates cautious interpretation [6, 94, 95]. Some studies have reported trends or statistically significant improvements in secondary endpoints [95, 96], including oxygenation and reductions in lung injury and inflammatory biomarkers such as SP-D, Ang-2, and IL-6 [69, 79, 84, 97]. Still, the impact of MSC therapy on ventilator duration and ICU stay remains uncertain [95].

To provide a comprehensive overview of clinical evidence, the key studies evaluating MSC and MSC-derived therapies for ARDS are summarized in Table 2 [10, 12, 30, 69, 75, 79, 84–86, 93, 96–104], highlighting study designs, MSC sources, dosing regimens, primary outcomes, efficacy, and safety profiles.

**Table 2:** Summary of clinical studies on MSCs and derived therapies for ARDS

First Author (Year)	Study Design	Sample Size Total (Treatment vs. Control)	MSC or Derived Therapy Source	ARDS Etiology	Dose and Administration Frequency	Main Outcomes	Efficacy	Safety
Zheng (2014) [69]	RCT	12 (6 vs. 6)	AD-MSC	ARDS (unspecified, PaO_2_/FiO_2_ < 200)	1 × 10⁶ cells/kg IV, single dose	Safety and lung injury biomarkers	Significant reduction in SP-D (day 5 vs. day 0, *P* = 0.027); Trend toward lower IL-6 (*P* = 0.06)	No infusion toxicities or SAEs; similar AEs in both groups; safe
Matthay (2019) [79]	RCT (Phase 2a START)	60 (40 vs. 20)	BM-MSC	Moderate-to-severe ARDS (Various non-COVID causes)	10 × 10⁶ cells/kg PBW IV, single dose	Safety	28-day mortality 30% (MSC) vs. 15% (Placebo) (OR 2.4, *P* = 0.58); No efficacy difference (adjusted HR 1.43, *P* = 0.58)	No predefined MSC-related AEs; 1 death in MSC group within 24 h (unrelated); safe
Sengupta (2020) [98]	Cohort	24 (Single arm)	BM-MSC-derived EVs	COVID-19 ARDS (Moderate-to-severe)	15 mL IV, single dose	Survival & oxygenation improvement	Survival 83% (17/24 recovered); PaO_2_/FiO_2_ increase 192% (*P* < 0.001); Reduced CRP (*P* < 0.001), ferritin (*P* < 0.001), D-dimer (*P* < 0.05)	No AEs within 72 h; safe
Hashemian (2021) [85]	Case Series	11 (Single arm)	UC-MSC, 6 cases) / PL-MSC, 5 cases	COVID-19 ARDS (requiring MV)	200 × 10⁶ cells IV, 3 doses (every other day)	Safety and clinical improvement	Clinical survival 6/11; Significant reduction in TNF-α (*P* < 0.01), IL-8 (*P* < 0.05), CRP (*P* < 0.01) in survivors	No SAEs reported 24–48 h post-infusion; safe
Lanzoni (2021) [10]	RCT (Phase 1/2a)	24 (12 vs. 12)	UC-MSC	COVID-19 ARDS	100 ± 20 × 10⁶ cells IV, 2 doses (Day 0 and 3)	Safety (AEs ≤ 6 h; cardiac arrest/death ≤ 24 h post-infusion), Patient survival at 31 days, Time to recovery	Patient survival 91% vs. 42% (*P* = 0.015); SAE-free survival (*P* = 0.008); Time to recovery (*P* = 0.03); Inflammatory cytokines significantly decreased at day 6	No difference in infusion-associated AEs; No SAEs related to UC-MSC; safe
Dilogo (2021) [99]	RCT	40 (20 vs. 20)	UC-MSC	COVID-19 ARDS (Critically ill)	1 × 10⁶ cells/kg IV, single dose	Survival rate, Length of ventilator usage	Survival rate 2.5 times higher in UC-MSC group (50% vs. 20%, *P* = 0.047); Significantly decreased IL-6 in recovered patients (*P* = 0.023)	No AEs reported; safe
Wick (2021) [97]	Nested Cohort (Matthay 2019)	27 (17 vs. 10)	BM-MSC	ARDS (Moderate-to-severe, mixed etiologies (e.g., pneumonia, sepsis))	10 × 10⁶ cells/kg IV, single dose	Airspace biomarker changes	Significantly reduced airspace total protein, Ang-2, IL-6, sTNFR1 vs. placebo; Airspace Ang-2 correlated with fewer VFD (*P* = 0.023)	(Safety reported in parent trial [Matthay 2019]); presumed safe [79]
Kouroupis (2021) [30]	Cohort	24 (from Lanzoni 2021 trial)	UC-MSC	COVID-19 ARDS	100 ± 20 × 10⁶ cells IV, 2 doses (Day 0 and 3)	Plasma TNFα, TNFβ, and sTNFR2 levels at Day 6	Significantly increased sTNFR2; Significantly decreased TNFα and TNFβ in UC-MSC group vs. controls (*P*-values not specified)	(Safety per Lanzoni 2021); presumed safe [10]
Monsel (2022) [100]	RCT (Phase 2b STROMA-CoV-2)	45 (21 vs. 24)	UC-MSC	COVID-19 ARDS (< 96 h)	1 × 10⁶ cells/kg IV, 3 infusions over 5 days	PaO_2_/FiO_2_ change (Day 0 to Day 7)	No significant difference in PaO_2_/FiO_2_ change (medians 54.3 vs. 25.3, *P* = 0.77)	SAEs 28.6% (UC-MSC) vs. 25% (Placebo), none related to treatment; safe
Farkhad (2022) [86]	RCT (Phase 1)	20 (10 vs. 10)	UC-MSC	COVID-19 ARDS (Mild-moderate)	1 × 10⁶ cells/kg IV, 3 doses (every other day)	Safety and respiratory function	Significant improvement in SpO_2_/FiO_2_ ratio; Significant decrease in CRP, IL-6, IFN-γ, TNF-α, IL-17 A (*P* < 0.05)	No serious AEs after cell transplantations; safe
Aghayan (2022) [75]	RCT (Phase 1)	20 (10 vs. 10)	PL-MSC	COVID-19 ARDS (ICU patients)	1 × 10⁶ cells/kg IV, single dose	Safety	No significant differences in hospital stay, SpO_2_, or other clinical/lab parameters (*P* >0.05)	No AEs observed in PL-MSC group; safe
Grégoire (2022) [87]	Cohort (vs. matched controls)	8 (MSC) vs. 24 (Matched Controls)	BM-MSC	COVID-19 ARDS (Severe, requiring O_2_ support)	1.5-3 × 10⁶ cells/kg IV, 3 infusions (at 3-day intervals)	Safety and survival	Survival at 28 & 60 days: 100% vs. 79.2% (*P* = 0.025) & 70.8% (*P* = 0.0082); Significantly lower Day-7 D-dimer in MSC group (*P* = 0.0085)	No AEs related to MSC infusions; safe
Bowdish (2023) [93]	RCT	222 (112 vs. 110)	Allogeneic MSC (Remestemcel-L)	COVID-19 ARDS (Moderate-to-severe)	2 × 10⁶ cells/kg IV, 2 infusions (Days 0, 4 ± 1)	30-day mortality	30-day mortality 37.5% vs. 42.7% (RR 0.88, *P* = 0.43); No significant differences in VFD	No infusion-related toxicities; similar SAEs; safe
Lightner (2023) [12]	RCT (Phase 2)	102 (Placebo vs. 10mL vs. EVs 15mL EVs; *N* ≈ 34 per arm)	BM-MSC-derived EVs (ExoFlo™)	COVID-19 ARDS (Moderate-to-severe)	Placebo, 10 mL, or 15 mL IV, 2 doses (Days 1 and 4)	All-cause 60-day mortality	Reduced 60-day mortality with 15 mL ExoFlo™ vs. placebo in post hoc subgroup (18–65 yrs, RR 0.385, *P* = 0.034); Improved VFD with 15 mL ExoFlo™ (18–65 yrs, *P* = 0.0455)	No treatment-related AEs; safe (15 mL dose)
Zarrabi (2023) [84]	RCT	43 (MSC:11, MSC + EV:8 vs. Control:24)	Unspecified source (MSC and MSC-EVs)	COVID-19 ARDS	MSC: 100 × 10⁶ cells IV, 2 doses; or MSC (100 × 10⁶) + EVs (1 dose each)	Mortality, Inflammatory markers	Mortality: MSC 3/11 (*P* = 0.08), MSC + EV 0/8 (*P* = 0.07) vs. control 8/24; MSC infusion associated with decreased IL-6 (*P* = 0.015), TNF-α (*P* = 0.034), IFN-γ (*P* = 0.024), CRP (*P* = 0.041)	No serious AEs; safe
Ichikado (2023) [101]	RCT (Phase 2, ONE-BRIDGE)	30 (20 vs. 10)	BM-derived Multipotent Adult Progenitor Cells	Pneumonia-induced ARDS (with early fibroproliferation)	9.0 × 10⁸ cells IV, single dose	VFDs through day 28	VFDs: 11.6 (Invimestrocel) vs. 6.2 (Standard) (LS mean difference 5.4, *P* = 0.1397); Mortality day 180: 26% vs. 43% (numerically lower with Invimestrocel)	No allergic or serious adverse reactions; well tolerated
Gorman (2023) [102]	RCT (REALIST-COVID)	60 (30 vs. 29)	UC-MSC (CD362+)	COVID-19 ARDS (Moderate-to-severe)	400 × 10⁶ cells IV, single dose	Day 7 oxygenation index, Incidence of SAEs	No difference in Day 7 oxygenation index (98.3 vs. 96.6); No differences in secondary outcomes or mortality	SAEs: 6 (ORBCEL-C) vs. 3 (Placebo) (RR 2.9, *P* = 0.25), none judged related to treatment; safe
Martínez-Muñoz (2024) [103]	RCT	20 (10 vs. 10)	BM-MSC	COVID-19 ARDS (Moderate-to-severe)	1 × 10⁶ cells/kg IV, single dose	Increase in PaO_2_/FiO_2_ ratio (Day 7)	No significant difference in PaO_2_/FiO_2_ (83.3 vs. 57.6); WHO score improved at day 7 (50% vs. 0%, *P* = 0.033); Shorter hospital stay (17.5 vs. 28 days, *P* = 0.042)	No infusion or treatment-related SAEs; safe at 1-year follow-up
Sitbon (2024) [104]	RCT (STROMA-CoV-2 long-term)	47 enrolled (19 completed 1-year FU)	UC-MSC	COVID-19 ARDS (from Monsel 2022)	1 × 10⁶ cells/kg IV, 3 doses (over 5 days)	Safety at 6 and 12 months	No significant differences in AEs, lung CT, PFTs, or QoL between groups at 6 & 12 months	No adverse effects observed at 1 year related to UC-MSC; favorable safety profile [100]

Abbreviations: RCT Randomized Controlled Trial; ARDS Acute Respiratory Distress Syndrome; MSC Mesenchymal Stromal Cells; EV Extracellular Vesicles; MAPC Multipotent Adult Progenitor Cells; SAE Serious Adverse Event; VFD Ventilator-Free Days; PBW Predicted Body Weight; IV Intravenous; MV Mechanical Ventilation; PEEP Positive End-Expiratory Pressure; SpO_2_/FiO_2_ Oxygen Saturation to Fraction of Inspired Oxygen Ratio; PaO_2_/FiO_2_ Partial Pressure of Oxygen to Fraction of Inspired Oxygen Ratio; CRP C-Reactive Protein; IL-6 Interleukin-6; TNF-α Tumor Necrosis Factor-alpha; IL-8 Interleukin-8; IFN-γ Interferon-gamma; IL-17 A Interleukin-17 A; Ang-2 Angiopoietin-2; sTNFR1 Soluble Tumor Necrosis Factor Receptor 1; sTNFR2 Soluble Tumor Necrosis Factor Receptor 2; SP-D Surfactant Protein-D; RR Relative Risk; HR Hazard Ratio; OR Odds Ratio; LS Least Squares; FU Follow-Up; QoL Quality of Life; PFTs Pulmonary Function Tests; CT Computed Tomography; WHO World Health Organization

### Cell-free MSC-derived therapies

Concerns regarding live cell transplantation—such as tumorigenicity, alloimmune responses, and embolism risks—as well as standardization challenges, have driven the exploration of cell-free therapies derived from MSC secretions. EVs, including exosomes and microvesicles, are nanosized vesicles that transport proteins, lipids, messenger RNAs (mRNAs), and microRNAs (miRNAs), and play essential roles in intercellular communication [105]. Their advantages encompass low immunogenicity, capacity to traverse biological barriers, absence of proliferative potential (reducing tumorigenic risk), and suitability for standardized production and storage [71, 98, 106]. Preclinical studies demonstrate that MSC-derived EVs can reduce inflammation, vascular permeability, and lung injury in ARDS models, often achieving therapeutic effects comparable to those of whole MSCs [7, 107–109], largely through transferring regulatory molecules like miRNAs (e.g., miR-223-3p [103], miR-27a-5p [104]) and mRNAs (e.g., Ang-1). Furthermore, preconditioning with agents such as interferon-γ (IFN-γ) [58], thrombin [110], or LPS [111], as well as genetic modifications, has been shown to augment EV therapeutic potency. In addition, nebulized administration of EVs has demonstrated superior anti-inflammatory effects in preclinical ARDS models [17, 112].

Clinically, Sengupta et al. demonstrated the safety and preliminary efficacy of ExoFlo™, a BM-MSC-derived EV product, in patients with severe COVID-19 [98]. In a randomized controlled trial, Lightner et al. observed a trend toward reduced mortality at specific dosages [12]. Moreover, Zarrabi et al. reported zero mortality in COVID-19 ARDS patients treated with a combination of MSCs and EVs [80, 84]. These early clinical findings highlight the potential of EV-based therapies, though larger, well-powered trials are warranted to confirm efficacy and safety.

Conditioned medium (CM) encompasses MSC-secreted soluble factors and EVs [25, 105]. Preclinical evidence indicates that CM, containing abundant cytokines (e.g., IL-10), growth factors (e.g., HGF), and EVs, can exert therapeutic effects comparable to MSCs in alleviating lung injury and improving pulmonary function [25, 105, 113, 114]. Similar to EVs, nebulized CM delivery has exhibited promising therapeutic outcomes in preclinical models, suggesting its potential as a noninvasive clinical administration route [76].

While preclinical studies highlight their anti-inflammatory and regenerative potential, direct clinical comparisons with whole MSCs are scarce. Ongoing trials (e.g., NCT03818854) are assessing the safety and efficacy of EVs in ARDS, and may yield crucial insights into their translational potential. Engineered artificial exosomes have shown promise in enhancing ALI treatment [109]. Advances in bioreactor-based culture systems and standardized analytical methods, such as nanoparticle tracking analysis, are being explored to improve reproducibility [115]. Thus, addressing these technical and regulatory hurdles is essential for translating exosome therapies into reliable clinical treatments for ARDS.

Despite their advantages in avoiding risks like embolism and tumorigenicity, MSC-derived exosomes face significant challenges in standardization and batch consistency. Variations in isolation methods (e.g., ultracentrifugation, size-exclusion chromatography), purification protocols, and characterization techniques can lead to inconsistent exosome yield, purity, and potency across batches. Furthermore, regulatory approval is hindered by their novel status and stringent safety requirements. These limitations pose major obstacles to the scalable clinical application of exosome-based therapies.

### Efficacy across ARDS subtypes

The heterogeneous nature of ARDS shapes MSC therapeutic responses across its etiologies and immunological profiles. Preclinical models, spanning LPS-induced, bacterial, viral, and ventilator-induced lung injury (VILI), consistently affirm MSC protective effects, albeit with varying degrees of responses [116]. However, the anti-fibrotic effects of MSCs may differ between pulmonary and extrapulmonary ARDS models [22]. In smoke inhalation ARDS models, concomitant burn injuries may compromise MSC efficacy by diverting their homing to cutaneous tissues, thus reducing pulmonary retention [117]. In models of ischemia-reperfusion injury, certain MSC subpopulations—such as multilineage-differentiating stress-enduring (Muse) cells—may exhibit superior therapeutic effects compared to those of conventional MSCs [118].

The COVID-19 pandemic significantly accelerated the clinical investigation of MSC therapy. Multiple RCTs and observational studies confirmed MSC safety, while meta-analyses suggested a potential reduction in mortality among COVID-19 ARDS. However, despite these encouraging findings, robust clinical evidence supporting MSC efficacy in non-COVID-19 ARDS remains limited [6, 94, 95, 119]. Although large-scale RCTs have not consistently achieved significant improvements in primary endpoints [96, 102], MSC safety is well-established, with some positive secondary outcome trends. Factors such as variations in control group treatments, baseline disease severity among enrolled patients, and differences in MSC preparation and administration protocols may help explain the discrepancies observed across clinical trials.

Furthermore, the underlying etiology of ARDS likely plays a crucial role in determining MSC therapeutic efficacy. Most recent clinical trials have focused on COVID-19-associated ARDS, which is characterized by a unique viral-induced endothelial injury and a specific cytokine storm profile. In this context, MSCs have shown promise in improving survival and reducing inflammatory markers in some studies [10, 99]. However, these findings may not be directly generalizable to ARDS from other causes, such as bacterial pneumonia, sepsis, or trauma, which involve different pathogenic mechanisms and immune responses. For instance, the ONE-BRIDGE trial specifically enrolled patients with pneumonia-induced ARDS and, while safe, did not reduce ventilator-free days [101]. This suggests that the therapeutic response to MSCs could be etiology-dependent. Future research must stratify results based on ARDS etiology to identify which patient populations are most likely to benefit and to tailor therapeutic strategies accordingly.

Distinct ARDS immunological phenotypes, including hyperinflammatory and hypoinflammatory subtypes, respond variably to MSC immunomodulatory effects [120]. Adjunctive approaches, such as antioxidant co-administration, have also been shown to amplify MSC therapeutic effects [121], emphasizing the need to tailor therapy to ARDS immunophenotypes.

Subgroup analyses of MSC trials in ARDS suggest that younger patients (aged < 65 years) may derive greater therapeutic benefit [96] possibly owing to their more robust immune responsiveness and intrinsic regenerative capacity [10, 96, 122], which may potentiate the immunomodulatory and reparative effects of MSCs. In addition to age, the host’s inflammatory phenotype plays a critical role in therapeutic response. For instance, a hyperinflammatory pulmonary milieu marked by elevated interleukin-6 (IL-6) levels may attenuate MSC efficacy [66], potentially by overwhelming their immunoregulatory mechanisms. Conversely, patients exhibiting specific cytokine profiles—such as lower baseline IL-6 or distinct soluble tumor necrosis factor receptor 2 (sTNFR2) concentrations—appear to have more favorable clinical outcomes [30]. These beneficial responses may be mediated, at least in part, by enhanced MSC-induced M2 macrophage polarization [20, 21] and expansion of regulatory Tregs [23]. Together, these findings highlight the relevance of age and inflammatory biomarkers (e.g., IL-6, sTNFR2) in patient stratification and support the development of biomarker-guided, personalized MSC therapies for ARDS.

Of note, these differences may be partially explained by age-dependent variations in MSC potency and the host inflammatory microenvironment (see Mechanisms section for details).

### Challenges and future directions

Despite their potential, MSC therapies encounter significant barriers to clinical implementation. The short-term safety of MSC therapy is firmly established [6, 67, 69, 79, 83], with one-year follow-up data showing no notable adverse events [104]. However, potential long-term risks—such as immunogenicity, tumorigenicity, and procoagulant effects—warrant ongoing surveillance and systematic evaluation [81].

#### Long-term safety and risks

MSCs and their derivatives generally exhibit low immunogenicity [53, 123], with AdMSC-derived exosomes and Muse cells specifically noted for lacking tumorigenicity [53, 118]. However, concerns about the potential tumorigenicity of parental MSCs [124] and their incomplete immunological privilege [125] persist, occasionally prompting strategies such as HLA downregulation to evade immune rejection [126]. Despite reassuring short-term findings, dedicated long-term monitoring is still needed to clarify risks such as tumorigenesis and immune responses following MSC therapy in ARDS survivors [125].

Limited follow-up duration remains a recognized challenge in this field. For instance, a systematic review by Kirkham et al. (2022) analyzing RCTs in COVID-19 ARDS reported a median follow-up of only 28 days across the included studies, underscoring the evidence gap for long-term outcomes [94]. Recent long-term follow-up (1–2 years) from clinical trials such as STROMA-CoV-2 and REALIST-COVID in ARDS patients has demonstrated favorable safety profiles for UC-MSCs, with no significant increase in adverse events or mortality compared to placebo [102, 104]. Nevertheless, a critical need remains for extended (e.g., ≥ 5 years) surveillance protocols within future trials to rigorously assess latent risks like tumorigenesis or chronic immunogenicity. Ongoing post-treatment surveillance will be essential to ensure long-term safety.

#### Inconsistency among trials and solutions

Inconsistent clinical trial outcomes, particularly concerning mortality, likely stem from variations in study design, patient diversity, and MSC properties. The dose-response relationship remains to be conclusively established and requires validation in larger-scale studies [79]. Key obstacles to clinical translation include non-standardized MSC characterization, culture, post-cryopreservation viability, batch consistency, and potency assays [13, 14, 127, 128]. To enhance efficacy, several strategies have been explored, including enriching functional subsets (e.g., CD362 + MSCs) [102, 129] and improvement of MSC homing and survival within inflamed microenvironments. Epigenetic HLA downregulation has been shown to reduce MSC immunogenicity [126], while gene modification [91, 92] and delivery optimization may help overcome these limitations.

#### Future directions

Future studies must prioritize refining multiple facets of MSC therapy, including cell source selection, dosing regimens, and administration timing. Advanced cellular engineering strategies also hold considerable promise. For instance, preconditioning approaches such as hypoxia exposure [70], or cytokine stimulation [130] can prime MSCs for enhanced function. Moreover, gene-editing technologies (e.g., CRISPR/Cas9) enable MSC modification to overexpress therapeutic mediators (Nrf2 [131], IL-10 [44], IL-35 [132]) or enhance homing properties via ACE2 [36], ROR2 [90], or AT2R [91], representing a frontier in MSC functional optimization.

In parallel, addressing manufacturing consistency is crucial. The development of controlled manufacturing platforms, such as microcarrier-based microbioreactors, can improve cell yield and standardize critical quality attributes (CQAs) far better than traditional flask cultures [128]. These advanced platforms, along with automated manufacturing systems, generate large, high-quality datasets suitable for artificial intelligence (AI) and machine learning models [133]. AI-based quality control holds transformative potential: for instance, deep learning models can non-invasively predict MSC functional potency and heterogeneity through live-cell microscopy or label-free spectral data [134–136]. This high-throughput, real-time method offers an alternative to conventional destructive assays, enhancing batch selection and therapeutic consistency.

Additionally, combination approaches—such as co-administration with antioxidants or glucocorticoids—warrant further investigation [137–139]. Parallel efforts should be directed toward the development and standardization of MSC-derived extracellular vesicles (EVs) and conditioned medium (CM), which represent promising cell-free alternatives. Priority should be given to conducting large-scale, multicenter RCTs (e.g., NCT03818854) and implementing adaptive clinical trial designs to rigorously assess the efficacy and safety of MSC-based therapies [94, 140].

Furthermore, exploring novel engineering strategies for MSCs, such as tailoring the MSC secretome or enhancing homing capacity, may help overcome translational barriers. Lastly, a critical innovative aspect for future research lies in dissecting the complex interplay between MSCs (or their derivatives) and the recipient’s immunophenotype and ARDS etiology. This understanding is paramount for biomarker-guided patient stratification and the realization of precision medicine approaches, enabling more targeted and effective MSC applications in ARDS [121].

## Conclusion

MSCs and their derivatives—including EVs and CM—exert multifaceted therapeutic effects in ARDS through mechanisms such as immunomodulation, anti-inflammation, antioxidation, and tissue repair. Accumulating preclinical studies and early-phase clinical trials, particularly those involving COVID-19-associated ARDS, have demonstrated their favorable safety profiles and suggested potential efficacy in specific patient subgroups. Nevertheless, the broad clinical translation of MSC-based therapies faces significant hurdles, including the need for standardized manufacturing, optimization of dosing and administration protocols, and precise patient stratification. To advance the field, high-quality RCTs and precision medicine–oriented strategies will be essential to fully realize the therapeutic potential of MSCs and facilitate their integration into routine clinical practice. Future research should not only focus on refining existing approaches but also on pioneering innovative strategies, such as advanced cell engineering and biomarker-driven personalized therapies, to truly harness the promise of MSCs for ARDS patients.

## Electronic Supplementary Material

Below is the link to the electronic supplementary material.

The original file of Figure 1

## Data Availability

No more data is available.

## Declarations

### Use of artificial intelligence (AI)

The authors declare that they have not used AI-generated work in this manuscript.

## References

[1] Lewis SR, Pritchard MW, Thomas CM, Smith AF. Pharmacological agents for adults with acute respiratory distress syndrome. Cochrane Database Syst Rev. 2019;7(7):CD004477. 31334568 10.1002/14651858.CD004477.pub3 PMC6646953

[2] Qadir N, Chang SY. Pharmacologic treatments for acute respiratory distress syndrome. Crit Care Clin. 2021;37(4):877–93. 34548139 10.1016/j.ccc.2021.05.009 PMC8449143

[3] Byrnes D, Masterson CH, Artigas A, Laffey JG. Mesenchymal stem/stromal cells therapy for sepsis and acute respiratory distress syndrome. Semin Respir Crit Care Med. 2021;42(1):20–39. 32767301 10.1055/s-0040-1713422

[4] Liang TY, Lu LH, Tang SY, Zheng ZH, Shi K, Liu JQ. Current status and prospects of basic research and clinical application of mesenchymal stem cells in acute respiratory distress syndrome. World J Stem Cells. 2023;15(4):150–64. 37180997 10.4252/wjsc.v15.i4.150 PMC10173811

[5] Simonson OE, Mougiakakos D, Heldring N, Bassi G, Johansson HJ, Dalen M, Jitschin R, Rodin S, Corbascio M, El Andaloussi S, et al. In vivo effects of mesenchymal stromal cells in two patients with severe acute respiratory distress syndrome. Stem Cells Transl Med. 2015;4(10):1199–213. 26285659 10.5966/sctm.2015-0021 PMC4572899

[6] Wang F, Li Y, Wang B, Li J, Peng Z. The safety and efficacy of mesenchymal stromal cells in ARDS: a meta-analysis of randomized controlled trials. Crit Care. 2023;27(1):31. 36670442 10.1186/s13054-022-04287-4 PMC9857915

[7] Wang F, Fang B, Qiang X, Shao J, Zhou L. The efficacy of mesenchymal stromal cell-derived therapies for acute respiratory distress syndrome-a meta-analysis of preclinical trials. Respir Res. 2020;21(1):307. 33218340 10.1186/s12931-020-01574-y PMC7677103

[8] Song M, Lv Q, Zhang X, Cao J, Sun S, Xiao P, Hou S, Ding H, Liu Z, Dong W et al. Dynamic Tracking Human Mesenchymal Stem Cells Tropism following Smoke Inhalation Injury in NOD/SCID Mice. Stem Cells Int. 2016;2016:1691856. 10.1155/2016/1691856 PMC5048056 27725837

[9] Isik S, Uzuner N, Karaman M, Karaman O, Kiray M, Kozanoglu I, Alper Bagriyanik H, Arikan-Ayyildiz Z, Kartal Yandim M, Baran Y. Effects of intraperitoneal injection of allogeneic bone Marrow-derived mesenchymal stem cells on bronchiolitis obliterans in mice model. Iran J Allergy Asthma Immunol. 2017;16(3):205–18. 28732434

[10] Lanzoni G, Linetsky E, Correa D, Messinger Cayetano S, Alvarez RA, Kouroupis D, Alvarez Gil A, Poggioli R, Ruiz P, Marttos AC, et al. Umbilical cord mesenchymal stem cells for COVID-19 acute respiratory distress syndrome: A double-blind, phase 1/2a, randomized controlled trial. Stem Cells Transl Med. 2021;10(5):660–73. 33400390 10.1002/sctm.20-0472 PMC8046040

[11] Regmi S, Ganguly A, Pathak S, Primavera R, Chetty S, Wang J, Patel S, Thakor AS. Evaluating the therapeutic potential of different sources of mesenchymal stem cells in acute respiratory distress syndrome. Stem Cell Res Ther. 2024;15(1):385. 39468662 10.1186/s13287-024-03977-w PMC11520775

[12] Lightner AL, Sengupta V, Qian S, Ransom JT, Suzuki S, Park DJ, Melson TI, Williams BP, Walsh JJ, Awili M. Bone marrow mesenchymal stem Cell-Derived extracellular vesicle infusion for the treatment of respiratory failure from COVID-19: A randomized, Placebo-Controlled dosing clinical trial. Chest. 2023;164(6):1444–53. 37356708 10.1016/j.chest.2023.06.024 PMC10289818

[13] Hao Q, Zhu YG, Monsel A, Gennai S, Lee T, Xu F, Lee JW. Study of bone marrow and embryonic stem Cell-Derived human mesenchymal stem cells for treatment of Escherichia coli Endotoxin-Induced acute lung injury in mice. Stem Cells Transl Med. 2015;4(7):832–40. 25999518 10.5966/sctm.2015-0006 PMC4479628

[14] Liao G, Zheng K, Lalu MM, Fergusson DA, Allan DS. A scoping review of registered clinical trials of cellular therapy for COVID-19 and a framework for accelerated synthesis of trial evidence-FAST evidence. Transfus Med Rev. 2020;34(3):165–71. 32684483 10.1016/j.tmrv.2020.06.001 PMC7320662

[15] Qin ZH, Xu JF, Qu JM, Zhang J, Sai Y, Chen CM, Wu L, Yu L. Intrapleural delivery of MSCs attenuates acute lung injury by paracrine/endocrine mechanism. J Cell Mol Med. 2012;16(11):2745–53. 22697354 10.1111/j.1582-4934.2012.01597.x PMC4118243

[16] Gupta N, Su X, Popov B, Lee JW, Serikov V, Matthay MA. Intrapulmonary delivery of bone marrow-derived mesenchymal stem cells improves survival and attenuates endotoxin-induced acute lung injury in mice. J Immunol. 2007;179(3):1855–63. 17641052 10.4049/jimmunol.179.3.1855

[17] Zhao R, Wang L, Wang T, Xian P, Wang H, Long Q. Inhalation of MSC-EVs is a noninvasive strategy for ameliorating acute lung injury. J Control Release. 2022;345:214–30. 35307508 10.1016/j.jconrel.2022.03.025

[18] Zhang TY, Zhang H, Deng JY, Gong HR, Yan Y, Zhang Z, Lei C. BMMSC-derived exosomes attenuate cardiopulmonary Bypass-related acute lung injury by reducing inflammatory response and oxidative stress. Curr Stem Cell Res Ther. 2023;18(5):720–8. 35996241 10.2174/1574888X17666220822123643

[19] Abdelmoneim M, El-Naenaeey EY, Abd-Allah SH, Gharib AA, Alhussein M, Aboalella DA, Abdelghany EM, Fathy MA, Hussein S. Anti-Inflammatory and Immunomodulatory role of bone Marrow-Derived MSCs in mice with acute lung injury. J Interferon Cytokine Res. 2021;41(1):29–36. 33471617 10.1089/jir.2020.0073

[20] Zhu J, Zhou J, Feng B, Pan Q, Yang J, Lang G, Shang D, Zhou J, Li L, Yu J, et al. MSCs alleviate LPS-induced acute lung injury by inhibiting the Proinflammatory function of macrophages in mouse lung organoid-macrophage model. Cell Mol Life Sci. 2024;81(1):124. 38466420 10.1007/s00018-024-05150-1 PMC10927843

[21] Hu Y, Qin C, Zheng G, Lai D, Tao H, Zhang Y, Qiu G, Ge M, Huang L, Chen L, et al. Mesenchymal stem Cell-Educated macrophages ameliorate LPS-Induced systemic response. Mediators Inflamm. 2016;2016:3735452. 27546994 10.1155/2016/3735452 PMC4978851

[22] Maron-Gutierrez T, Silva JD, Asensi KD, Bakker-Abreu I, Shan Y, Diaz BL, Goldenberg RC, Mei SH, Stewart DJ, Morales MM, et al. Effects of mesenchymal stem cell therapy on the time course of pulmonary remodeling depend on the etiology of lung injury in mice. Crit Care Med. 2013;41(11):e319–333. 23760104 10.1097/CCM.0b013e31828a663e

[23] Xue M, Zhang X, Chen J, Liu F, Xu J, Xie J, Yang Y, Yu W, Qiu H. Mesenchymal Stem Cell-Secreted TGF-beta1 Restores Treg/Th17 Skewing Induced by Lipopolysaccharide and Hypoxia Challenge via miR-155 Suppression. *Stem Cells Int* 2022, 2022:5522828. 10.1155/2022/5522828 PMC8934213 35313652

[24] Tian Z, Li Y, Ji P, Zhao S, Cheng H. Mesenchymal stem cells protects hyperoxia-induced lung injury in newborn rats via inhibiting receptor for advanced glycation end-products/nuclear factor kappab signaling. Exp Biol Med (Maywood). 2013;238(2):242–7. 23576805 10.1177/1535370212473706

[25] Tang Y, Ding F, Wu C, Liu B. hucMSC Conditioned Medium Ameliorate Lipopolysaccharide-Induced Acute Lung Injury by Suppressing Oxidative Stress and Inflammation via Nrf2/NF-kappaB Signaling Pathway. Anal Cell Pathol (Amst). 2021;2021:6653681. 10.1155/2021/6653681 PMC8380155 34426780

[26] Hua F, Cui E, Lv L, Wang B, Li L, Lu H, Chen N, Chen W. Fecal microbiota transplantation from HUC-MSC-treated mice alleviates acute lung injury in mice through anti-inflammation and gut microbiota modulation. Front Microbiol. 2023;14:1243102. 37840733 10.3389/fmicb.2023.1243102 PMC10569429

[27] Yang H, Liu Y, Yao J, Wang Y, Wang L, Ren P, Bai B, Wen Q. Mesenchymal stem cells inhibit ferroptosis by activating the Nrf2 antioxidation pathway in severe acute pancreatitis-associated acute lung injury. Eur J Pharmacol. 2024;967:176380. 38311279 10.1016/j.ejphar.2024.176380

[28] Zhang X, Wei X, Deng Y, Yuan X, Shi J, Huang W, Huang J, Chen X, Zheng S, Chen J, et al. Mesenchymal stromal cells alleviate acute respiratory distress syndrome through the cholinergic anti-inflammatory pathway. Signal Transduct Target Ther. 2022;7(1):307. 36064538 10.1038/s41392-022-01124-6 PMC9441842

[29] Hu X, Liu L, Wang Y, Yu Y, Li Z, Liu Y, Chai J. Human Umbilical Cord-Derived Mesenchymal Stem Cells Alleviate Acute Lung Injury Caused by Severe Burn via Secreting TSG-6 and Inhibiting Inflammatory Response. Stem Cells Int. 2022;2022:8661689. 10.1155/2022/8661689 PMC8881119 35222649

[30] Kouroupis D, Lanzoni G, Linetsky E, Messinger Cayetano S, Wishnek Metalonis S, Lenero C, Stone LD, Ruiz P, Correa D, Ricordi C. Umbilical Cord-derived mesenchymal stem cells modulate TNF and soluble TNF receptor 2 (sTNFR2) in COVID-19 ARDS patients. Eur Rev Med Pharmacol Sci. 2021;25(12):4435–8. 34227081 10.26355/eurrev_202106_26156

[31] Xu M, Li X, Ma C, Lu Y, Ma X, Ma X. [Effect of human placental mesenchymal stem cells transplantation on pulmonary vascular endothelial permeability and lung injury repair in mice with acute lung injury]. Zhongguo Xiu Fu Chong Jian Wai Ke Za Zhi. 2020;34(3):387–92. 32174088 10.7507/1002-1892.201909070 PMC8171659

[32] Fang X, Neyrinck AP, Matthay MA, Lee JW. Allogeneic human mesenchymal stem cells restore epithelial protein permeability in cultured human alveolar type II cells by secretion of angiopoietin-1. J Biol Chem. 2010;285(34):26211–22. 20554518 10.1074/jbc.M110.119917 PMC2924032

[33] Matthay MA. Therapeutic potential of mesenchymal stromal cells for acute respiratory distress syndrome. Ann Am Thorac Soc. 2015;12(Suppl 1Suppl 1):S54–57. 25830837 10.1513/AnnalsATS.201406-254MG PMC4430975

[34] Nakajima D, Watanabe Y, Ohsumi A, Pipkin M, Chen M, Mordant P, Kanou T, Saito T, Lam R, Coutinho R, et al. Mesenchymal stromal cell therapy during ex vivo lung perfusion ameliorates ischemia-reperfusion injury in lung transplantation. J Heart Lung Transpl. 2019;38(11):1214–23. 10.1016/j.healun.2019.07.006 31474491

[35] Yang Y, Hu S, Xu X, Li J, Liu A, Han J, Liu S, Liu L, Qiu H. The vascular endothelial growth Factors-Expressing character of mesenchymal stem cells plays a positive role in treatment of acute lung injury in vivo. Mediators Inflamm. 2016;2016:2347938. 27313398 10.1155/2016/2347938 PMC4895047

[36] Min F, Gao F, Li Q, Liu Z. Therapeutic effect of human umbilical cord mesenchymal stem cells modified by angiotensin-converting enzyme 2 gene on bleomycin-induced lung fibrosis injury. Mol Med Rep. 2015;11(4):2387–96. 25435005 10.3892/mmr.2014.3025 PMC4337478

[37] Lee SH, Jang AS, Kim YE, Cha JY, Kim TH, Jung S, Park SK, Lee YK, Won JH, Kim YH, et al. Modulation of cytokine and nitric oxide by mesenchymal stem cell transfer in lung injury/fibrosis. Respir Res. 2010;11(1):16. 20137099 10.1186/1465-9921-11-16 PMC2827393

[38] Gong L, Wang X, Xu S, Liao F, Zhou M. Human Amnion-Derived MSCs Alleviate Acute Lung Injury and Hinder Pulmonary Fibrosis Caused by Paraquat in Rats. Oxid Med Cell Longev 2022;2022:3932070. 10.1155/2022/3932070 PMC8957415 35345827

[39] Cai Y, Zou Z, Liu L, Chen S, Chen Y, Lin Z, Shi K, Xu L, Chen Y. Bone marrow-derived mesenchymal stem cells inhibits hepatocyte apoptosis after acute liver injury. Int J Clin Exp Pathol. 2015;8(1):107–16. 25755697 PMC4348892

[40] Zhang X, Gao F, Li Q, Dong Z, Sun B, Hou L, Li Z, Liu Z. MSCs with ACE II gene affect apoptosis pathway of acute lung injury induced by bleomycin. Exp Lung Res. 2015;41(1):32–43. 25419634 10.3109/01902148.2014.963901

[41] Shalaby SM, El-Shal AS, Abd-Allah SH, Selim AO, Selim SA, Gouda ZA, Abd El Motteleb DM, Zanfaly HE, El-Assar HM, Abdelazim S. Mesenchymal stromal cell injection protects against oxidative stress in Escherichia coli-induced acute lung injury in mice. Cytotherapy. 2014;16(6):764–75. 24525173 10.1016/j.jcyt.2013.12.006

[42] Iyer SS, Torres-Gonzalez E, Neujahr DC, Kwon M, Brigham KL, Jones DP, Mora AL, Rojas M. Effect of bone marrow-derived mesenchymal stem cells on endotoxin-induced oxidation of plasma cysteine and glutathione in mice. Stem Cells Int 2010;2010:868076. 10.4061/2010/868076 PMC2963315 21048855

[43] Horie S, Masterson C, Brady J, Loftus P, Horan E, O’Flynn L, Elliman S, Barry F, O’Brien T, Laffey JG, et al. Umbilical cord-derived CD362(+) mesenchymal stromal cells for E. coli pneumonia: impact of dose regimen, passage, cryopreservation, and antibiotic therapy. Stem Cell Res Ther. 2020;11(1):116. 32169108 10.1186/s13287-020-01624-8 PMC7071745

[44] Jerkic M, Masterson C, Ormesher L, Gagnon S, Goyal S, Rabani R, Otulakowski G, Zhang H, Kavanagh BP, Laffey JG. Overexpression of IL-10 enhances the efficacy of human Umbilical-Cord-Derived mesenchymal stromal cells in E. coli Pneumosepsis. J Clin Med 2019;8(6). 10.3390/jcm8060847 PMC6616885 31200579

[45] Li L, Dong L, Gao F, Hui J, Chen Y, Yan J. [Effects of Hippo signaling pathway on lung injury repair by mesenchymal stem cells in acute respiratory distress syndrome]. Zhonghua Wei Zhong Bing Ji Jiu Yi Xue. 2019;31(3):281–7. 30914086 10.3760/cma.j.issn.2095-4352.2019.03.005

[46] Morrison TJ, Jackson MV, Cunningham EK, Kissenpfennig A, McAuley DF, O’Kane CM, Krasnodembskaya AD. Mesenchymal stromal cells modulate macrophages in clinically relevant lung injury models by extracellular vesicle mitochondrial transfer. Am J Respir Crit Care Med. 2017;196(10):1275–86. 28598224 10.1164/rccm.201701-0170OC PMC5694830

[47] Cardenes N, Caceres E, Romagnoli M, Rojas M. Mesenchymal stem cells: a promising therapy for the acute respiratory distress syndrome. Respiration. 2013;85(4):267–78. 23428562 10.1159/000347072

[48] Jackson MV, Morrison TJ, Doherty DF, McAuley DF, Matthay MA, Kissenpfennig A, O’Kane CM, Krasnodembskaya AD. Mitochondrial transfer via tunneling nanotubes is an important mechanism by which mesenchymal stem cells enhance macrophage phagocytosis in the in vitro and in vivo models of ARDS. Stem Cells. 2016;34(8):2210–23. 27059413 10.1002/stem.2372 PMC4982045

[49] Zhang K, Gao Y, Deng Y, Zhou X, Zhu C, He Z, Lv D. Studies on the effects of bone marrow stem cells on mitochondrial function and the alleviation of ARDS. Mol Cell Biochem. 2021;476(1):93–107. 32845436 10.1007/s11010-020-03888-3 PMC7447610

[50] Dutra Silva J, Su Y, Calfee CS, Delucchi KL, Weiss D, McAuley DF, O’Kane C, Krasnodembskaya AD. Mesenchymal stromal cell extracellular vesicles rescue mitochondrial dysfunction and improve barrier integrity in clinically relevant models of ARDS. Eur Respir J 2021;58(1). 10.1183/13993003.02978-2020 PMC8318599 33334945

[51] Wang J, Meng S, Chen Y, Wang H, Hu W, Liu S, Huang L, Xu J, Li Q, Wu X, et al. MSC-mediated mitochondrial transfer promotes metabolic reprograming in endothelial cells and vascular regeneration in ARDS. Redox Rep. 2025;30(1):2474897. 40082392 10.1080/13510002.2025.2474897 PMC11912292

[52] Zhang F, Zheng X, Zhao F, Li L, Ren Y, Li L, Huang H, Yin H. TFAM-Mediated mitochondrial transfer of MSCs improved the permeability barrier in sepsis-associated acute lung injury. Apoptosis. 2023;28(7–8):1048–59. 37060506 10.1007/s10495-023-01847-z

[53] Xia L, Zhang C, Lv N, Liang Z, Ma T, Cheng H, Xia Y, Shi L. AdMSC-derived exosomes alleviate acute lung injury via transferring mitochondrial component to improve homeostasis of alveolar macrophages. Theranostics. 2022;12(6):2928–47. 35401830 10.7150/thno.69533 PMC8965475

[54] Zhou X, Zhang K, He Z, Deng Y, Gao Y. Downregulated miR-150 in bone marrow mesenchymal stem cells attenuates the apoptosis of LPS-stimulated RAW264.7 via MTCH2-dependent mitochondria transfer. Biochem Biophys Res Commun. 2020;526(3):560–7. 32247615 10.1016/j.bbrc.2020.03.098

[55] Zhang M, Xu G, Zhou X, Luo M, Ma N, Wang X, Wang Z, Tang H, Wang X, Li Y, et al. Mesenchymal stem cells ameliorate H9N2-induced acute lung injury by inhibiting caspase-3-GSDME-mediated pyroptosis of lung alveolar epithelial cells. Eur J Pharmacol. 2023;960:176148. 37866742 10.1016/j.ejphar.2023.176148

[56] Lv L, Cui EH, Wang B, Li LQ, Hua F, Lu HD, Chen N, Chen WY. Multiomics reveal human umbilical cord mesenchymal stem cells improving acute lung injury via the lung-gut axis. World J Stem Cells. 2023;15(9):908–30. 37900940 10.4252/wjsc.v15.i9.908 PMC10600741

[57] Sun J, Ding X, Liu S, Duan X, Liang H, Sun T. Adipose-derived mesenchymal stem cells attenuate acute lung injury and improve the gut microbiota in septic rats. Stem Cell Res Ther. 2020;11(1):384. 32894198 10.1186/s13287-020-01902-5 PMC7487801

[58] Varkouhi AK, Jerkic M, Ormesher L, Gagnon S, Goyal S, Rabani R, Masterson C, Spring C, Chen PZ, Gu FX, et al. Extracellular vesicles from Interferon-gamma-primed human umbilical cord mesenchymal stromal cells reduce Escherichia coli-induced acute lung injury in rats. Anesthesiology. 2019;130(5):778–90. 30870158 10.1097/ALN.0000000000002655

[59] Fathi E, Charoudeh HN, Sanaat Z, Farahzadi R. Telomere shortening as a hallmark of stem cell senescence. Stem Cell Investig. 2019;6:7. 31019963 10.21037/sci.2019.02.04 PMC6458335

[60] Dexheimer V, Mueller S, Braatz F, Richter W. Reduced reactivation from dormancy but maintained lineage choice of human mesenchymal stem cells with donor age. PLoS ONE. 2011;6(8):e22980. 21850247 10.1371/journal.pone.0022980 PMC3151268

[61] Kretlow JD, Jin YQ, Liu W, Zhang WJ, Hong TH, Zhou G, Baggett LS, Mikos AG, Cao Y. Donor age and cell passage affects differentiation potential of murine bone marrow-derived stem cells. BMC Cell Biol. 2008;9:60. 18957087 10.1186/1471-2121-9-60 PMC2584028

[62] Stolzing A, Jones E, McGonagle D, Scutt A. Age-related changes in human bone marrow-derived mesenchymal stem cells: consequences for cell therapies. Mech Ageing Dev. 2008;129(3):163–73. 18241911 10.1016/j.mad.2007.12.002

[63] Scruggs BA, Semon JA, Zhang X, Zhang S, Bowles AC, Pandey AC, Imhof KM, Kalueff AV, Gimble JM, Bunnell BA. Age of the donor reduces the ability of human adipose-derived stem cells to alleviate symptoms in the experimental autoimmune encephalomyelitis mouse model. Stem Cells Transl Med. 2013;2(10):797–807. 24018793 10.5966/sctm.2013-0026 PMC3785264

[64] Gnani D, Crippa S, Della Volpe L, Rossella V, Conti A, Lettera E, Rivis S, Ometti M, Fraschini G, Bernardo ME, et al. An early-senescence state in aged mesenchymal stromal cells contributes to hematopoietic stem and progenitor cell clonogenic impairment through the activation of a pro-inflammatory program. Aging Cell. 2019;18(3):e12933. 30828977 10.1111/acel.12933 PMC6516180

[65] Liang H, Hou H, Yi W, Yang G, Gu C, Lau WB, Gao E, Ma X, Lu Z, Wei X, et al. Increased expression of pigment epithelium-derived factor in aged mesenchymal stem cells impairs their therapeutic efficacy for attenuating myocardial infarction injury. Eur Heart J. 2013;34(22):1681–90. 21606086 10.1093/eurheartj/ehr131 PMC3675387

[66] Islam D, Huang Y, Fanelli V, Delsedime L, Wu S, Khang J, Han B, Grassi A, Li M, Xu Y, et al. Identification and modulation of microenvironment is crucial for effective mesenchymal stromal cell therapy in acute lung injury. Am J Respir Crit Care Med. 2019;199(10):1214–24. 30521764 10.1164/rccm.201802-0356OC

[67] Wilson JG, Liu KD, Zhuo H, Caballero L, McMillan M, Fang X, Cosgrove K, Vojnik R, Calfee CS, Lee JW, et al. Mesenchymal stem (stromal) cells for treatment of ARDS: a phase 1 clinical trial. Lancet Respir Med. 2015;3(1):24–32. 25529339 10.1016/S2213-2600(14)70291-7 PMC4297579

[68] Lu H, Cook T, Poirier C, Merfeld-Clauss S, Petrache I, March KL, Bogatcheva NV. Pulmonary retention of adipose stromal cells following intravenous delivery is markedly altered in the presence of ARDS. Cell Transpl. 2016;25(9):1635–43. 10.3727/096368915X690189 26609693

[69] Zheng G, Huang L, Tong H, Shu Q, Hu Y, Ge M, Deng K, Zhang L, Zou B, Cheng B, et al. Treatment of acute respiratory distress syndrome with allogeneic adipose-derived mesenchymal stem cells: a randomized, placebo-controlled pilot study. Respir Res. 2014;15(1):39. 24708472 10.1186/1465-9921-15-39 PMC3994204

[70] Gorman E, Millar J, McAuley D, O’Kane C. Mesenchymal stromal cells for acute respiratory distress syndrome (ARDS), sepsis, and COVID-19 infection: optimizing the therapeutic potential. Expert Rev Respir Med. 2021;15(3):301–24. 33172313 10.1080/17476348.2021.1848555

[71] Alzahrani FA, Saadeldin IM, Ahmad A, Kumar D, Azhar EI, Siddiqui AJ, Kurdi B, Sajini A, Alrefaei AF, Jahan S. The Potential Use of Mesenchymal Stem Cells and Their Derived Exosomes as Immunomodulatory Agents for COVID-19 Patients. Stem Cells Int 2020;2020:8835986. 10.1155/2020/8835986 PMC7512102 33014070

[72] Rodriguez HC, Gupta M, Cavazos-Escobar E, El-Amin SF 3rd, Gupta A. Umbilical cord: an allogenic tissue for potential treatment of COVID-19. Hum Cell. 2021;34(1):1–13. 33033884 10.1007/s13577-020-00444-5 PMC7544522

[73] Nie Z, Fan Q, Jiang W, Wei S, Luo R, Hu H, Liu G, Lei Y, Xie S. Placental mesenchymal stem cells suppress inflammation and promote M2-like macrophage polarization through the IL-10/STAT3/NLRP3 axis in acute lung injury. Front Immunol. 2024;15:1422355. 39620220 10.3389/fimmu.2024.1422355 PMC11604576

[74] Xu M, Liu G, Jia Y, Yan X, Ma X, Wei J, Liu X, Ma X. [Transplantation of human placenta mesenchymal stem cells reduces the level of inflammatory factors in lung tissues of mice with acute lung injury]. Xi Bao Yu Fen Zi Mian Yi Xue Za Zhi. 2018;34(2):105–9. 29673451

[75] Aghayan HR, Salimian F, Abedini A, Fattah Ghazi S, Yunesian M, Alavi-Moghadam S, Makarem J, Majidzadeh AK, Hatamkhani A, Moghri M, et al. Human placenta-derived mesenchymal stem cells transplantation in patients with acute respiratory distress syndrome (ARDS) caused by COVID-19 (phase I clinical trial): safety profile assessment. Stem Cell Res Ther. 2022;13(1):365. 35902979 10.1186/s13287-022-02953-6 PMC9330663

[76] Gonzalez HE, McCarthy SD, Masterson C, Laffey JG, MacLoughlin R, O’Toole D. Nebulized mesenchymal stem cell derived conditioned medium ameliorates Escherichia coli induced pneumonia in a rat model. Front Med (Lausanne). 2023;10:1162615. 37332742 10.3389/fmed.2023.1162615 PMC10272576

[77] Curley GF, Ansari B, Hayes M, Devaney J, Masterson C, Ryan A, Barry F, O’Brien T, Toole DO, Laffey JG. Effects of intratracheal mesenchymal stromal cell therapy during recovery and resolution after ventilator-induced lung injury. Anesthesiology. 2013;118(4):924–32. 23377221 10.1097/ALN.0b013e318287ba08

[78] Kwan TN, Zwakman-Hessels L, Marhoon N, Robbins R, Martensson J, Ekinci E, Bellomo R. Relative hypoglycemia in diabetic patients with critical illness. Crit Care Med. 2020;48(3):e233–40. 31876532 10.1097/CCM.0000000000004213

[79] Matthay MA, Calfee CS, Zhuo H, Thompson BT, Wilson JG, Levitt JE, Rogers AJ, Gotts JE, Wiener-Kronish JP, Bajwa EK, et al. Treatment with allogeneic mesenchymal stromal cells for moderate to severe acute respiratory distress syndrome (START study): a randomised phase 2a safety trial. Lancet Respir Med. 2019;7(2):154–62. 30455077 10.1016/S2213-2600(18)30418-1 PMC7597675

[80] Tieu A, Stewart DJ, Chwastek D, Lansdell C, Burger D, Lalu MM. Biodistribution of mesenchymal stromal cell-derived extracellular vesicles administered during acute lung injury. Stem Cell Res Ther. 2023;14(1):250. 37705086 10.1186/s13287-023-03472-8 PMC10500845

[81] Yigenoglu TN, Basci S, Sahin D, Ulas T, Dal MS, Korkmaz S, Hacibekiroglu T, Namdaroglu S, Erkurt MA, Turgut B, et al. Mesenchymal stem cell transfusion: possible beneficial effects in COVID-19 patients. Transfus Apher Sci. 2021;60(6):103237. 34419356 10.1016/j.transci.2021.103237 PMC8372452

[82] Chen X, Shan Y, Wen Y, Sun J, Du H. Mesenchymal stem cell therapy in severe COVID-19: A retrospective study of short-term treatment efficacy and side effects. J Infect. 2020;81(4):647–79. 32422152 10.1016/j.jinf.2020.05.020 PMC7227593

[83] Sanchez-Guijo F, Garcia-Arranz M, Lopez-Parra M, Monedero P, Mata-Martinez C, Santos A, Sagredo V, Alvarez-Avello JM, Guerrero JE, Perez-Calvo C, et al. Adipose-derived mesenchymal stromal cells for the treatment of patients with severe SARS-CoV-2 pneumonia requiring mechanical ventilation. A proof of concept study. EClinicalMedicine. 2020;25:100454. 32838232 10.1016/j.eclinm.2020.100454 PMC7348610

[84] Zarrabi M, Shahrbaf MA, Nouri M, Shekari F, Hosseini SE, Hashemian SR, Aliannejad R, Jamaati H, Khavandgar N, Alemi H, et al. Allogenic mesenchymal stromal cells and their extracellular vesicles in COVID-19 induced ARDS: a randomized controlled trial. Stem Cell Res Ther. 2023;14(1):169. 37365605 10.1186/s13287-023-03402-8 PMC10294333

[85] Hashemian SR, Aliannejad R, Zarrabi M, Soleimani M, Vosough M, Hosseini SE, Hossieni H, Keshel SH, Naderpour Z, Hajizadeh-Saffar E, et al. Mesenchymal stem cells derived from perinatal tissues for treatment of critically ill COVID-19-induced ARDS patients: a case series. Stem Cell Res Ther. 2021;12(1):91. 33514427 10.1186/s13287-021-02165-4 PMC7844804

[86] Kaffash Farkhad N, Sedaghat A, Reihani H, Adhami Moghadam A, Bagheri Moghadam A, Khadem Ghaebi N, Khodadoust MA, Ganjali R, Tafreshian AR, Tavakol-Afshari J. Mesenchymal stromal cell therapy for COVID-19-induced ARDS patients: a successful phase 1, control-placebo group, clinical trial. Stem Cell Res Ther. 2022;13(1):283. 35765103 10.1186/s13287-022-02920-1 PMC9241239

[87] Liu G, Di Z, Hao C, Wang W, Pei T, Zheng L, Long H, Wang H, Liao W, Wang W, et al. Effects of different concentrations of mesenchymal stem cells treatment on LPS-induced acute respiratory distress syndrome rat model. Exp Lung Res. 2021;47(5):226–38. 33749474 10.1080/01902148.2021.1897191

[88] Lin KC, Fang WF, Sung PH, Huang KT, Chiang JY, Chen YL, Huang CR, Li YC, Lee MS, Yip HK. Early and Dose-Dependent xenogeneic mesenchymal stem cell therapy improved outcomes in acute respiratory distress syndrome rodent through ameliorating inflammation, oxidative stress, and immune reaction. Cell Transpl. 2023;32:9636897231190178. 10.1177/09636897231190178 PMC10469224 37592717

[89] Tai WL, Dong ZX, Zhang DD, Wang DH. Therapeutic effect of intravenous bone marrow-derived mesenchymal stem cell transplantation on early-stage LPS-induced acute lung injury in mice. Nan Fang Yi Ke Da Xue Xue Bao. 2012;32(3):283–90. 22445968

[90] Cai SX, Liu AR, Chen S, He HL, Chen QH, Xu JY, Pan C, Yang Y, Guo FM, Huang YZ, et al. The orphan receptor tyrosine kinase ROR2 facilitates MSCs to repair lung injury in ARDS animal model. Cell Transpl. 2016;25(8):1561–74. 10.3727/096368915X689776 26531175

[91] Xu XP, Huang LL, Hu SL, Han JB, He HL, Xu JY, Xie JF, Liu AR, Liu SQ, Liu L, et al. Genetic modification of mesenchymal stem cells overexpressing angiotensin II type 2 receptor increases cell migration to injured lung in LPS-Induced acute lung injury mice. Stem Cells Transl Med. 2018;7(10):721–30. 30133167 10.1002/sctm.17-0279 PMC6186265

[92] Xu L, Shen JM, Qu JL, Song N, Che XF, Hou KZ, Shi J, Zhao L, Shi S, Liu YP, et al. FEN1 is a prognostic biomarker for ER + breast cancer and associated with Tamoxifen resistance through the eralpha/cyclin D1/Rb axis. Ann Transl Med. 2021;9(3):258. 33708885 10.21037/atm-20-3068 PMC7940940

[93] Gregoire C, Layios N, Lambermont B, Lechanteur C, Briquet A, Bettonville V, Baudoux E, Thys M, Dardenne N, Misset B, et al. Bone Marrow-Derived mesenchymal stromal cell therapy in severe COVID-19: preliminary results of a phase I/II clinical trial. Front Immunol. 2022;13:932360. 35860245 10.3389/fimmu.2022.932360 PMC9291273

[94] Kirkham AM, Bailey AJM, Shorr R, Lalu MM, Fergusson DA, Allan DS. Systematic review and meta-analysis of randomized controlled trials of mesenchymal stromal cells to treat coronavirus disease 2019: is it too late? Cytotherapy 2023;25(3):341–52. 10.1016/j.jcyt.2022.10.003 PMC9556962 36333234

[95] Fang L, Hu F, Li H, Chang W, Liu L. Efficacy and safety of mesenchymal stem cell therapy for acute respiratory distress syndrome-a systematic review and meta-analysis. J Thorac Dis. 2024;16(9):5802–14. 39444918 10.21037/jtd-24-281 PMC11494583

[96] Bowdish ME, Barkauskas CE, Overbey JR, Gottlieb RL, Osman K, Duggal A, Marks ME, Hupf J, Fernandes E, Leshnower BG, et al. A randomized trial of mesenchymal stromal cells for moderate to severe acute respiratory distress syndrome from COVID-19. Am J Respir Crit Care Med. 2023;207(3):261–70. 36099435 10.1164/rccm.202201-0157OC PMC9896641

[97] Wick KD, Leligdowicz A, Zhuo H, Ware LB, Matthay MA. Mesenchymal stromal cells reduce evidence of lung injury in patients with ARDS. JCI Insight 2021;6(12). 10.1172/jci.insight.148983 PMC8262503 33974564

[98] Sengupta V, Sengupta S, Lazo A, Woods P, Nolan A, Bremer N. Exosomes derived from bone marrow mesenchymal stem cells as treatment for severe COVID-19. Stem Cells Dev. 2020;29(12):747–54. 32380908 10.1089/scd.2020.0080 PMC7310206

[99] Dilogo IH, Aditianingsih D, Sugiarto A, Burhan E, Damayanti T, Sitompul PA, Mariana N, Antarianto RD, Liem IK, Kispa T, et al. Umbilical cord mesenchymal stromal cells as critical COVID-19 adjuvant therapy: A randomized controlled trial. Stem Cells Transl Med. 2021;10(9):1279–87. 34102020 10.1002/sctm.21-0046 PMC8242692

[100] Monsel A, Hauw-Berlemont C, Mebarki M, Heming N, Mayaux J, Nguekap Tchoumba O, Diehl JL, Demoule A, Annane D, Marois C, et al. Treatment of COVID-19-associated ARDS with mesenchymal stromal cells: a multicenter randomized double-blind trial. Crit Care. 2022;26(1):48. 35189925 10.1186/s13054-022-03930-4 PMC8860258

[101] Ichikado K, Kotani T, Kondoh Y, Imanaka H, Johkoh T, Fujimoto K, Nunomiya S, Kawayama T, Sawada M, Jenkins E, et al. Clinical efficacy and safety of multipotent adult progenitor cells (invimestrocel) for acute respiratory distress syndrome (ARDS) caused by pneumonia: a randomized, open-label, standard therapy-controlled, phase 2 multicenter study (ONE-BRIDGE). Stem Cell Res Ther. 2023;14(1):217. 37608287 10.1186/s13287-023-03451-z PMC10464414

[102] Gorman EA, Rynne J, Gardiner HJ, Rostron AJ, Bannard-Smith J, Bentley AM, Brealey D, Campbell C, Curley G, Clarke M, et al. Repair of acute respiratory distress syndrome in COVID-19 by stromal cells (REALIST-COVID Trial): A Multicenter, Randomized, Controlled Clinical Trial. Am J Respir Crit Care Med. 2023;208(3):256–69. 37154608 10.1164/rccm.202302-0297OC

[103] Martinez-Munoz ME, Payares-Herrera C, Lipperheide I, Malo de Molina R, Salcedo I, Alonso R, Martin-Donaire T, Sanchez R, Zafra R, Garcia-Berciano M, et al. Mesenchymal stromal cell therapy for COVID-19 acute respiratory distress syndrome: a double-blind randomised controlled trial. Bone Marrow Transpl. 2024;59(6):777–84. 10.1038/s41409-024-02230-5 38409332

[104] Sitbon A, Hauw-Berlemont C, Mebarki M, Heming N, Mayaux J, Diehl JL, Demoule A, Annane D, Marois C, Demeret S, et al. Treatment of COVID-19-associated ARDS with umbilical cord-derived mesenchymal stromal cells in the STROMA-CoV-2 multicenter randomized double-blind trial: long-term safety, respiratory function, and quality of life. Stem Cell Res Ther. 2024;15(1):109. 38637891 10.1186/s13287-024-03729-w PMC11027516

[105] Muraca M, Pessina A, Pozzobon M, Dominici M, Galderisi U, Lazzari L, Parolini O, Lucarelli E, Perilongo G, Baraldi E. Mesenchymal stromal cells and their secreted extracellular vesicles as therapeutic tools for COVID-19 pneumonia? J Control Release. 2020;325:135–40. 32622963 10.1016/j.jconrel.2020.06.036 PMC7332437

[106] Rezakhani L, Kelishadrokhi AF, Soleimanizadeh A, Rahmati S. Mesenchymal stem cell (MSC)-derived exosomes as a cell-free therapy for patients infected with COVID-19: real opportunities and range of promises. Chem Phys Lipids. 2021;234:105009. 33189639 10.1016/j.chemphyslip.2020.105009 PMC7658620

[107] Tieu A, Hu K, Gnyra C, Montroy J, Fergusson DA, Allan DS, Stewart DJ, Thebaud B, Lalu MM. Mesenchymal stromal cell extracellular vesicles as therapy for acute and chronic respiratory diseases: A meta-analysis. J Extracell Vesicles. 2021;10(12):e12141. 34596349 10.1002/jev2.12141 PMC8485337

[108] Khatri M, Richardson LA, Meulia T. Mesenchymal stem cell-derived extracellular vesicles attenuate influenza virus-induced acute lung injury in a pig model. Stem Cell Res Ther. 2018;9(1):17. 29378639 10.1186/s13287-018-0774-8 PMC5789598

[109] Liang L, Peng W, Qin A, Zhang J, Chen R, Zhou D, Zhang X, Zhou N, Yu XY, Zhang L. Intracellularly synthesized artificial exosome treats acute lung injury. ACS Nano. 2024;18(32):21009–23. 39087239 10.1021/acsnano.4c01900

[110] Bang Y, Hwang S, Kim YE, Sung DK, Yang M, Ahn SY, Sung SI, Joo KM, Chang YS. Therapeutic efficacy of thrombin-preconditioned mesenchymal stromal cell-derived extracellular vesicles on Escherichia coli-induced acute lung injury in mice. Respir Res. 2024;25(1):303. 39112999 10.1186/s12931-024-02908-w PMC11308396

[111] Areny-Balaguero A, Camprubi-Rimblas M, Campana-Duel E, Sole-Porta A, Ceccato A, Roig A, Laffey JG, Closa D, Artigas A. Priming mesenchymal stem cells with lipopolysaccharide boosts the Immunomodulatory and regenerative activity of secreted extracellular vesicles. Pharmaceutics 2024, 16(10). 10.3390/pharmaceutics16101316 PMC11510928 39458645

[112] Wang J, Chen ZJ, Zhang ZY, Shen MP, Zhao B, Zhang W, Zhang Y, Lei JG, Ren CJ, Chang J, et al. Manufacturing, quality control, and GLP-grade preclinical study of nebulized allogenic adipose mesenchymal stromal cells-derived extracellular vesicles. Stem Cell Res Ther. 2024;15(1):95. 38566259 10.1186/s13287-024-03708-1 PMC10988864

[113] Emukah C, Dittmar E, Naqvi R, Martinez J, Corral A, Moreira A, Moreira A. Mesenchymal stromal cell conditioned media for lung disease: a systematic review and meta-analysis of preclinical studies. Respir Res. 2019;20(1):239. 31666086 10.1186/s12931-019-1212-x PMC6822429

[114] Moreira A, Naqvi R, Hall K, Emukah C, Martinez J, Moreira A, Dittmar E, Zoretic S, Evans M, Moses D, et al. Effects of mesenchymal stromal cell-conditioned media on measures of lung structure and function: a systematic review and meta-analysis of preclinical studies. Stem Cell Res Ther. 2020;11(1):399. 32933584 10.1186/s13287-020-01900-7 PMC7493362

[115] Ulpiano C, Salvador W, Franchi-Mendes T, Huang MC, Lin YH, Lin HT, Rodrigues CAV, Fernandes-Platzgummer A, Cabral JMS, Monteiro GA, et al. Continuous collection of human mesenchymal-stromal-cell-derived extracellular vesicles from a stirred tank reactor operated under xenogeneic-free conditions for therapeutic applications. Stem Cell Res Ther. 2025;16(1):210. 40275409 10.1186/s13287-025-04341-2 PMC12023423

[116] Tunstead C, Volkova E, Dunbar H, Hawthorne IJ, Bell A, Crowe L, Masterson JC, Dos Santos CC, McNicholas B, Laffey JG, et al. The ARDS microenvironment enhances MSC-induced repair via VEGF in experimental acute lung inflammation. Mol Ther. 2024;32(10):3422–32. 39108095 10.1016/j.ymthe.2024.08.003 PMC11489539

[117] Baljinnyam T, Radnaa E, Niimi Y, Fukuda S, Prough DS, Enkhbaatar P. Cutaneous burn diminishes beneficial effect of intravenously administered mesenchymal stem cells on acute lung injury induced by smoke inhalation in sheep. Burns. 2020;46(8):1914–23. 32513501 10.1016/j.burns.2020.05.012 PMC11676003

[118] Yabuki H, Watanabe T, Oishi H, Katahira M, Kanehira M, Okada Y. Muse cells and Ischemia-Reperfusion lung injury. Adv Exp Med Biol. 2018;1103:293–303. 30484236 10.1007/978-4-431-56847-6_16

[119] Qu W, Wang Z, Engelberg-Cook E, Yan D, Siddik AB, Bu G, Allickson JG, Kubrova E, Caplan AI, Hare JM, et al. Efficacy and safety of MSC cell therapies for hospitalized patients with COVID-19: A systematic review and Meta-Analysis. Stem Cells Transl Med. 2022;11(7):688–703. 35640138 10.1093/stcltm/szac032 PMC9299515

[120] Nanchal RS, Truwit JD. Recent advances in understanding and treating acute respiratory distress syndrome. F1000Res 2018;7. 10.12688/f1000research.15493.1 PMC6107983 30210781

[121] Zhang H, Li Y, Slutsky AS. Precision medicine for cell therapy in acute respiratory distress syndrome. Lancet Respir Med. 2019;7(4):e13. 30926050 10.1016/S2213-2600(19)30089-X

[122] Jaukovic A, Abadjieva D, Trivanovic D, Stoyanova E, Kostadinova M, Pashova S, Kestendjieva S, Kukolj T, Jeseta M, Kistanova E, et al. Specificity of 3D MSC spheroids microenvironment: impact on MSC behavior and properties. Stem Cell Rev Rep. 2020;16(5):853–75. 32681232 10.1007/s12015-020-10006-9

[123] Liu C, Xiao K, Xie L. Advances in mesenchymal stromal cell therapy for acute lung injury/acute respiratory distress syndrome. Front Cell Dev Biol. 2022;10:951764. 36036014 10.3389/fcell.2022.951764 PMC9399751

[124] Cheng Y, Cao X, Qin L. Mesenchymal stem Cell-Derived extracellular vesicles: A novel Cell-Free therapy for sepsis. Front Immunol. 2020;11:647. 32373121 10.3389/fimmu.2020.00647 PMC7186296

[125] Chen W, Lv L, Chen N, Cui E. Immunogenicity of mesenchymal stromal/stem cells. Scand J Immunol. 2023;97(6):e13267. 39007962 10.1111/sji.13267

[126] Wang F, Li R, Xu JY, Bai X, Wang Y, Chen XR, Pan C, Chen S, Zhou K, Heng BC, et al. Downregulating human leucocyte antigens on mesenchymal stromal cells by epigenetically repressing a beta(2)-microglobulin super-enhancer. Nat Biomed Eng. 2024;8(12):1682–99. 39433971 10.1038/s41551-024-01264-w

[127] Duong A, Parmar G, Kirkham AM, Burger D, Allan DS. Registered clinical trials investigating treatment with cell-derived extracellular vesicles: a scoping review. Cytotherapy. 2023;25(9):939–45. 37191614 10.1016/j.jcyt.2023.04.007

[128] Krupczak B, Farruggio C, Van Vliet KJ. Manufacturing mesenchymal stromal cells in a microcarrier-microbioreactor platform can enhance cell yield and quality attributes: case study for acute respiratory distress syndrome. J Transl Med. 2024;22(1):614. 38956643 10.1186/s12967-024-05373-7 PMC11220991

[129] Sundberg M, Bogetofte H, Lawson T, Jansson J, Smith G, Astradsson A, Moore M, Osborn T, Cooper O, Spealman R, et al. Improved cell therapy protocols for parkinson’s disease based on differentiation efficiency and safety of hESC-, hiPSC-, and non-human primate iPSC-derived dopaminergic neurons. Stem Cells. 2013;31(8):1548–62. 23666606 10.1002/stem.1415 PMC3775937

[130] Horie S, Gaynard S, Murphy M, Barry F, Scully M, O’Toole D, Laffey JG. Cytokine pre-activation of cryopreserved xenogeneic-free human mesenchymal stromal cells enhances resolution and repair following ventilator-induced lung injury potentially via a KGF-dependent mechanism. Intensive Care Med Exp. 2020;8(1):8. 32025852 10.1186/s40635-020-0295-5 PMC7002627

[131] Xu L, Zhu Y, Li C, Wang Q, Ma L, Wang J, Zhang S. Small extracellular vesicles derived from Nrf2-overexpressing human amniotic mesenchymal stem cells protect against lipopolysaccharide-induced acute lung injury by inhibiting NLRP3. Biol Direct. 2022;17(1):35. 36447296 10.1186/s13062-022-00351-9 PMC9706911

[132] Zhang X, Zhang Z, Ju M, Li J, Jing Y, Zhao Y, Gu C, Dong M, Li G, Liu Y. Pretreatment with Interleukin 35-engineered mesenchymal stem cells protected against lipopolysaccharide-induced acute lung injury via pulmonary inflammation suppression. Inflammopharmacology. 2020;28(5):1269–81. 32170527 10.1007/s10787-020-00696-5 PMC7095386

[133] Zhu K, Ding Y, Chen Y, Su K, Zheng J, Zhang Y, Hu Y, Wei J, Wang Z. Advancing regenerative medicine: the Aceman system’s pioneering automation and machine learning in mesenchymal stem cell biofabrication. Biofabrication 2025;17(2). 10.1088/1758-5090/adb803 39970480

[134] Imboden S, Liu X, Lee BS, Payne MC, Hsieh CJ, Lin NYC. Investigating heterogeneities of live mesenchymal stromal cells using AI-based label-free imaging. Sci Rep. 2021;11(1):6728. 33762607 10.1038/s41598-021-85905-z PMC7991643

[135] Kim G, Jeon JH, Park K, Kim SW, Kim DH, Lee S. High throughput screening of mesenchymal stem cell lines using deep learning. Sci Rep. 2022;12(1):17507. 36266301 10.1038/s41598-022-21653-y PMC9584889

[136] Wang J, Luo Y, Wu Y, Du F, Shi S, Duan Y, Chen A, Zhang J, Yu S. Single-cell Raman spectroscopy as a novel platform for unveiling the heterogeneity of mesenchymal stem cells. Talanta. 2025;292:127933. 40081243 10.1016/j.talanta.2025.127933

[137] Newton CA, Zhang D, Oldham JM, Kozlitina J, Ma SF, Martinez FJ, Raghu G, Noth I, Garcia CK. Telomere length and use of immunosuppressive medications in idiopathic pulmonary fibrosis. Am J Respir Crit Care Med. 2019;200(3):336–47. 30566847 10.1164/rccm.201809-1646OC PMC6680304

[138] Yang H, Wen Y, Hou-you Y, Yu-tong W, Chuan-ming L, Jian X, Lu H. Combined treatment with bone marrow mesenchymal stem cells and Methylprednisolone in paraquat-induced acute lung injury. BMC Emerg Med. 2013;13(Suppl 1Suppl 1):S5. 23902576 10.1186/1471-227X-13-S1-S5 PMC3701473

[139] Feng Y, Yang XT, Wang LL, Qu JM, Song YL. [Effect of adipose-derived mesenchymal stem cells and liraglutide on acute lung injury]. Zhonghua Jie He He Hu Xi Za Zhi. 2020;43(9):765–71. 32894910 10.3760/cma.j.cn112147-20200621-00733

[140] Kirkham AM, Monaghan M, Bailey AJM, Shorr R, Lalu MM, Fergusson DA, Allan DS. Mesenchymal stem/stromal cell-based therapies for COVID-19: first iteration of a living systematic review and meta-analysis: MSCs and COVID-19. Cytotherapy. 2022;24(6):639–49. 35219584 10.1016/j.jcyt.2021.12.001 PMC8802614

