# Supplementary figures and images for: Mesenchymal stem cell therapies for ARDS: translational promise and challenges
Source: Stem Cell Res Ther. 2025 Sep 26;16:504. doi: 10.1186/s13287-025-04614-w (PMC12465782; doi:10.1186/s13287-025-04614-w)

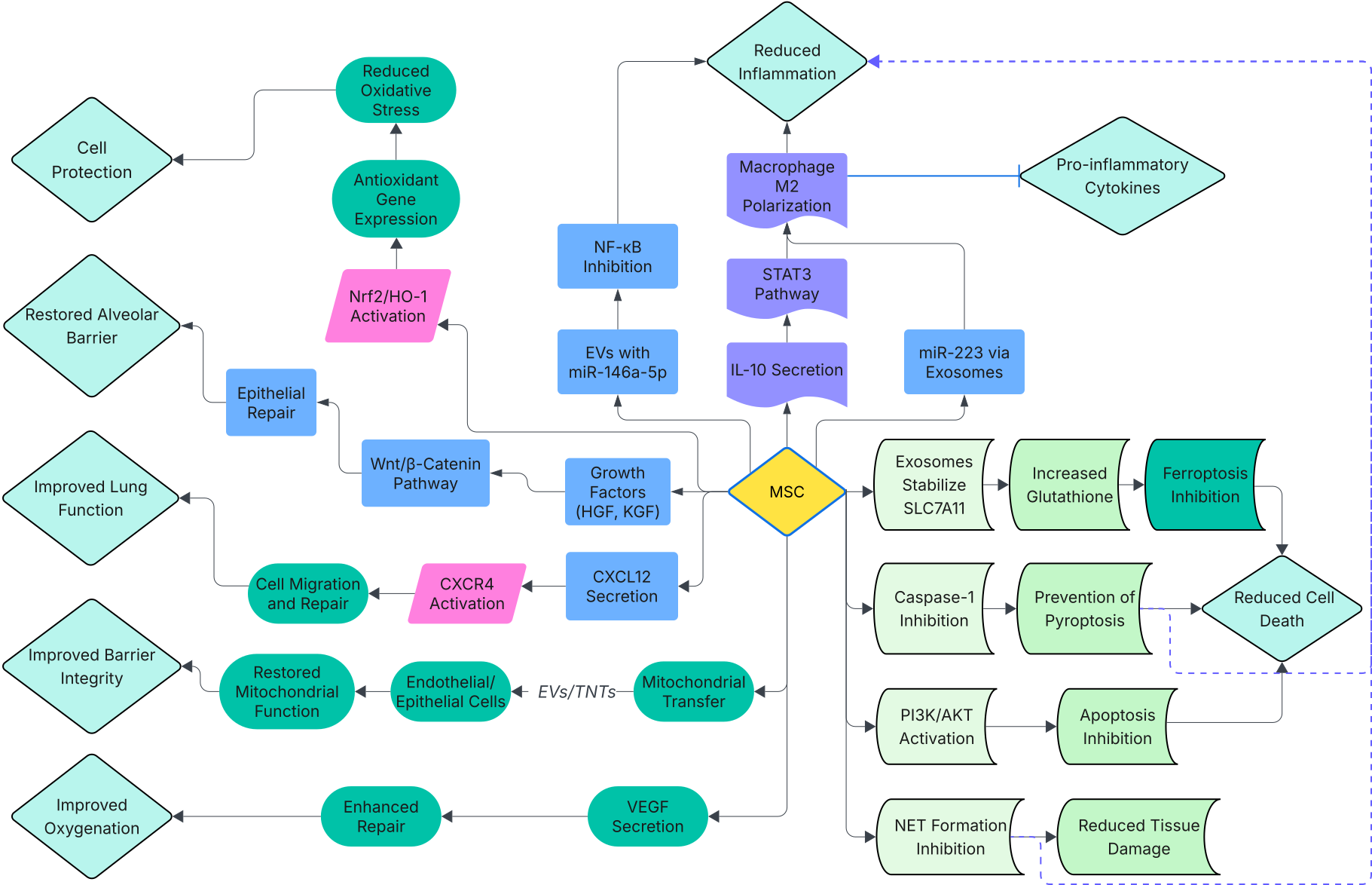

Supplement: Supplementary file 1 — The original file of Figure 1 [file 13287_2025_4614_MOESM1_ESM.png]
